# Cutting-Edge: Preclinical and Clinical Development of the First Approved Lag-3 Inhibitor

**DOI:** 10.3390/cells11152351

**Published:** 2022-07-30

**Authors:** Luisa Chocarro, Ana Bocanegra, Ester Blanco, Leticia Fernández-Rubio, Hugo Arasanz, Miriam Echaide, Maider Garnica, Pablo Ramos, Sergio Piñeiro-Hermida, Ruth Vera, David Escors, Grazyna Kochan

**Affiliations:** 1 Oncoimmunology Research Unit, Navarrabiomed-Fundación Miguel Servet, Universidad Pública de Navarra (UPNA), Hospital Universitario de Navarra (HUN), Instituto de Investigación Sanitaria de Navarra (IdiSNA), 31001 Pamplona, Spain; eblancop@navarra.es (E.B.); lfernanr@navarra.es (L.F.-R.); harasane@navarra.es (H.A.); mechaidg@navarra.es (M.E.); mgarnics@navarra.es (M.G.); pramosca@navarra.es (P.R.); sergio.pineiro.hermida@navarra.es (S.P.-H.); descorsm@navarra.es (D.E.); grkochan@navarra.es (G.K.); 2 Division of Gene Therapy and Regulation of Gene Expression, Cima Universidad de Navarra, Instituto de Investigación Sanitaria de Navarra (IdISNA), 31001 Pamplona, Spain; 3 Medical Oncology Unit, Hospital Universitario de Navarra (HUN), Instituto de Investigación Sanitaria de Navarra (IdiSNA), 31001 Pamplona, Spain; ruth.vera.garcia@navarra.es

**Keywords:** opdualag, relatimab, BMS-986016, nivolumab, LAG-3, PD-1

## Abstract

Immune checkpoint inhibitors (ICIs) have revolutionized medical practice in oncology since the FDA approval of the first ICI 11 years ago. In light of this, Lymphocyte-Activation Gene 3 (LAG-3) is one of the most important next-generation immune checkpoint molecules, playing a similar role as Programmed cell Death protein 1 (PD-1) and Cytotoxic T-Lymphocyte Antigen 4 (CTLA-4). 19 LAG-3 targeting molecules are being evaluated at 108 clinical trials which are demonstrating positive results, including promising bispecific molecules targeting LAG-3 simultaneously with other ICIs. Recently, a new dual anti-PD-1 (Nivolumab) and anti-LAG-3 (Relatimab) treatment developed by Bristol Myers Squibb (Opdualag), was approved by the Food and Drug Administration (FDA) as the first LAG-3 blocking antibody combination for unresectable or metastatic melanoma. This novel immunotherapy combination more than doubled median progression-free survival (PFS) when compared to nivolumab monotherapy (10.1 months versus 4.6 months). Here, we analyze the large clinical trial responsible for this historical approval (RELATIVITY-047), and discuss the preclinical and clinical developments that led to its jump into clinical practice. We will also summarize results achieved by other LAG-3 targeting molecules with promising anti-tumor activities currently under clinical development in phases I, I/II, II, and III. Opdualag will boost the entry of more LAG-3 targeting molecules into clinical practice, supporting the accumulating evidence highlighting the pivotal role of LAG-3 in cancer.

## 1. Introduction: Brief History of Immunotherapy

The use of monoclonal antibodies blocking T-cell inhibitory receptors has enormously revolutionized the treatment of various haematological and solid cancers, thanks to the reinvigoration of endogenous antitumor immune responses. Since its early beginnings, immunotherapy has rapidly evolved, leading to durable clinical responses in patients with advanced-stage tumors. Nowadays, immune checkpoint blockade (ICB) constitutes a standard clinical therapy, even as a first-line choice. Up to date, nine immune checkpoint inhibitors (ICIs) targeting Programmed cell Death protein 1 (PD-1), Programmed cell death ligand 1 (PD-L1), Cytotoxic T-Lymphocyte Antigen 4 (CTLA-4) and Lymphocyte-Activation Gene 3 (LAG-3) receptors have been approved for clinical use. Despite its enormous potential, ICB immunotherapy still presents a variety of drawbacks that remain to be addressed. The failure to respond in a significant number of patients is probably the main one. This failure can be attributed to tumor refractoriness, acquired resistance, and deleterious immune-related adverse events. Aiming to increase the efficacy of ICB monotherapies, several combinatorial approaches have been developed. The rationale behind these combinations is the targeting of differential mechanisms exerted by distinct checkpoint receptors and their corresponding ligands in the tumor microenvironment. This co-targeting is thought to trigger synergistic immune responses in terms of increased progression-free survival (PFS) and reduced number of unresponsive patients. On 18 March 2022, the Food and Drug Administration (FDA) approved one of these combinatorial immunotherapies. Under the name of Opdualag, the combination of the LAG-3 and PD-1 ICIs relatlimab and nivolumab gained authorization for use in adult and some paediatric patients with unresectable or metastatic melanoma. Here, we will contextualize the clinical trial that brought this therapy to the market, RELATIVITY-047. We will review the pathway of Opdualag towards its approval. In addition, we will summarize the results achieved by the other LAG-3 targeting molecules currently at phase I, I/II, II and III, and review the preclinical and clinical development of other ‘second generation’ ICIs that may follow the steps of LAG-3 in a near future.

## 2. Preclinical and Clinical Development of Opdualag

### 2.1. LAG-3 Molecular Function

The LAG-3 molecule has recently emerged as a promising cancer immunotherapy target and a highly important next-generation immune checkpoint molecule [1,2,3]. Closely related to CD4, and adjacent to its locus, it presents a similar genetic organization [4]. LAG-3 was first described as an immune inhibitory receptor in activated T cells. It plays a similar role than its immune-checkpoint counterparts PD-1 and CTLA-4 [5,6]. LAG-3 exerts an inhibitory function over multiple biological functions, such as T cell activation, immune function, proliferation, cytokine secretion, effector functions and T cell homeostasis [5,6,7,8,9]. For example, LAG-3 regulates the size of the expanding T cell population following antigen activation in vivo [8]. In broad terms, LAG-3 down-modulates TCR:CD3 intracellular signal transduction cascades and calcium fluxes within the immunological synapse, terminating cytokine and T cell responses to the TCR:CD3 activation, while favouring CD4 and CD8 T cell exhaustion [8,10,11,12,13,14,15].

LAG-3 is expressed by many T cell subsets, including: CD4 T helper cells, cytotoxic CD8 T cells, activated T cells, NK T cells, effector CD4 T cells, regulatory T cells, CD8 tumor-infiltrating lymphocytes and tumor-infiltrating antigen-specific CD8 T cells [4,16,17,18,19,20,21,22,23,24,25,26,27]. However, LAG-3 expression is not limited to T cells, having been described in B cells, natural regulatory plasma cells or B cells, plasmacytoid dendritic cells (DCs) and in neurons among others [15,27,28,29,30,31]. 

Initially, its high-affinity binding with the class II Major Histocompatibility Complex (MHC-II) was considered to mediate its inhibitory functions. MHC-II was thought to be its canonical ligand. MHC-II binds to LAG-3 with higher affinity than CD4, thus inhibiting CD4 T cell activation by competition with its binding to CD4 [5,6,32,33,34,35,36]. However, while it is undeniable that LAG-3:MHC-II interaction plays a critical role, LAG-3 binding to other ligands contributes to its inhibitory activities. The next functional ligands to be described were galectin-3 (Gal-3), critical to inhibit T cell activation and CD8 cytotoxic T cell functions [37,38,39,40], the liver-secreted protein fibrinogen-like protein 1 (FGL1), critical for tumor immune evasion mechanisms in response to anti-PD-1/anti-PD-L1 treatments [9,37,41,42,43], and the DC-specific Intercellular adhesion molecule-3-grabbing non-integrin family member (LSECtin) in melanoma cells, inhibiting cyclin-dependent kinases [44].

### 2.2. LAG-3 Clinical Research

LAG-3 is generally considered an aggressive progression marker in several haematological and solid malignancies, driving T cell exhaustion and pro-apoptosis, and associated with poor prognosis and decreased survival. Its expression is also considered an intrinsic resistance mechanism to anti-PD-1/anti-PD-L1 therapies through its synergic co-expression with PD-1 [12,22,45,46,47,48,49,50,51,52]. For example, non-small cell lung cancer (NSCLC) patients who did not respond to anti-PD-L1/PD-1 monotherapies had highly dysfunctional T cells that strongly co-expressed PD-1 and LAG-3 after TCR stimulation [22]. ICB therapies, especially anti-PD-1/anti-PD-L1 treatments, have revolutionized cancer treatment in recent years. However, not all patients respond to treatment due to intrinsic or extrinsic resistance mechanisms [1]. LAG-3 over-expression confers resistance to PD-1 blockade. Indeed, PD-1/LAG-3 co-blockade is demonstrating encouraging results and strong capacities both in preclinical and clinical research [2]. In this context, LAG-3-targeted therapies have emerged as a cancer immunotherapy alone and in combination with anti-PD-1 treatments. A new generation of novel bispecific molecules is being evaluated with encouraging results in preclinical and clinical studies.

**Figure 1:**
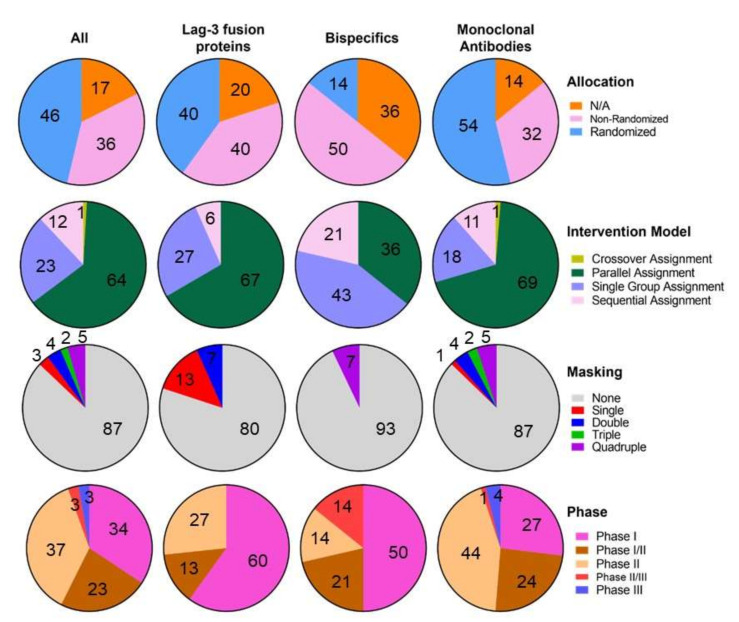
LAG-3-targeted therapy clinical landscape allocation, intervention models and masking of LAG-3 targeted molecules including LAG-3 fusion proteins, bispecific molecules and monoclonal antibodies (https://clinicaltrials.gov/, accessed on 29 June 2022). Percentages are indicated within the graphs.

**Figure 2:**
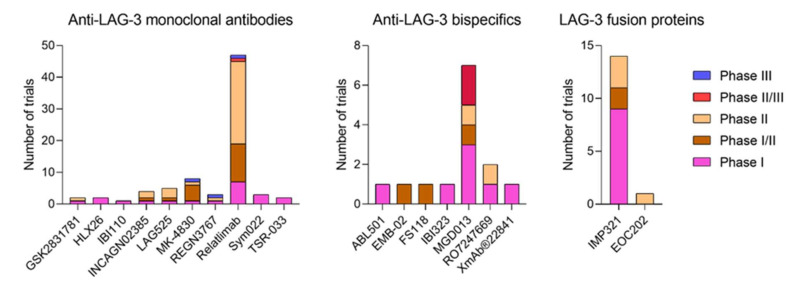
LAG-3-targeted therapy clinical landscape of phases for anti-LAG-3 monoclonal antibodies, bispecific molecules and fusion proteins clinically developed (https://clinicaltrials.gov/, accessed on 29 June 2022).

108 interventional clinical trials are evaluating 19 different LAG-3 targeting molecules in 39 phase I trials, 2 phase I/II trials, 40 phase II trials, 3 phase II/III trials and 3 phase III trials (Tables S1–S4). These molecules can be divided into anti-LAG-3 monoclonal antibodies (178 trials, 10 molecules), bispecific molecules (14 trials, 7 molecules), LAG-3 fusion proteins (15 trials, 2 molecules) and CAR-T cells (1 trial, 1 molecule). In total, more than 28,000 adult patients are being enrolled with the exception of the NCT03470922 phase II/III trial, which is enrolling patients of 12 years old patients and older. A total of 23 trials are active but not recruiting, 16 are completed, 8 not yet recruiting, 49 recruiting, 9 terminated and 2 withdrawn. Only 13 have available results. According to allocation, 50 trials are randomized, 39 non-randomized, and 19 N/A. On the intervention model, 68 trials follow a parallel assignment, 1 a crossover assignment, 25 a single group assignment and 13 a sequential assignment. Most of them follow an open label masking, while three of them a single (participant), four a double (participant and investigator), two a triple (participant, care provider and investigator) and four a quadruple (participant, care provider, investigator and outcomes assessor) (Figure 1 and Figure 2). Treated neoplasias include most hematological and solid cancers, but also psoriasis, ulcerative colitis and influenza.

Interestingly, LAG-3 expression is associated with increased pathology and impaired immune responses in multiples diseases such as Parkinson’s Disease [29,30], cardiovascular diseases (increased coronary heart disease and increased myocardial infarction) [53,54], HDL Hypercholesterolemia [55,56], Inflammatory Bowel Disease [57,58], Multiple Sclerosis [59], Diabetes Mellitus [60,61] and infection (Salmonella [31], Plasmodium parasites (P. yoelii 17XL, P yoelii 17XNL, P. chabaudi, P. vinckei, and P. berghei) [62], Mycobacterium tuberculosis [63], human immunodeficiency virus (HIV) [64], non-pathogenic simian immunodeficiency virus (SIV) [65], hepatitis B virus (HBV) [66], human papillomavirus (HPV) [67], hepatitis C virus (HCV) [67], lymphocytic choriomeningitis viral (LCMV), herpes simplex virus 1 (HSV-1) and other chronic viral infections [15,68,69,70,71,72]. Thus, LAG-3 targeted strategies currently under clinical development for cancer will also be relevant as immunotherapies for the treatment of non-neoplastic diseases [73,74,75,76,77].

#### 2.2.1. Anti-LAG-3 Monoclonal Antibodies

78 clinical trials are evaluating 10 different anti-LAG-3 monoclonal antibodies: BMS-986016 or relatlimab (Bristol-Myers Squibb), GSK2831781 (GlaxoSmithKline), HLX26 (Fosun Pharma), IBI110 (Innovent Biologics), INCAGN02385 (Incyte), LAG525 or IMP701 (Novartis), MK-4830 or favezelimab (Merck), REGN3767 or fianlimab (Regeneron Pharmaceuticals and Sanofi), Sym022 (Symphogen), TSR-033 (Tesaro) (Table S1). Of these, 21 are phase I, 19 phase I/II, 24 phase II, 1 phase II/III and 3 phase III. 7 phase I and II trials are only evaluating the anti-LAG-3 treatment alone (NCT05078593, NCT03489369, NCT03965533, NCT02195349, NCT05039658, NCT03538028, NCT03893565), 10 phase I, I/II and III trials are evaluating the anti-LAG-3 treatment alone and in combination with other ICIs (NCT02658981, NCT03250832, NCT03005782, NCT02966548, NCT02720068, NCT04150965, NCT01968109, NCT02061761, NCT02460224, NCT03743766) and the rest are evaluating anti-LAG-3 treatments in combination with other treatments, mainly with additional ICIs such as anti-PD-1. Most of them are fully humanized IgG4 blocking antibodies.

- **BMS-986016** or **relatlimab**, developed by Bristol-Myers Squibb in 47 clinical trials, was the first anti-LAG-3 monoclonal antibody to be clinically developed and the first one to receive the FDA approval for its clinical use. It has 4 subunits, with 16 disulfide links and 2 N-glycosylation sites, with an average molecular weight of 145.3 kDa [78]. Phase I (7 trials), I/II (12 trials), II (26 trials), II/III (1 trial) and III (1 trial) preliminary results showed good tolerability, efficacy, toxicity and antitumour profiles alone or in combination with anti-PD-1/PD-L1 blockade immunotherapies, as a good alternative to overcome immunotherapy resistance [79,80,81]. For example, it restores T cell mediated responses and TNF-a, IFN-y and IL-2 cytokine release [82]. The phase III clinical trial that led to the LAG-3/PD-1 combination approval for melanoma treatment is further discussed in the next section.
- **GSK2831781**, derived from **IMP731** Immunetep’s antibody, developed in monotherapy by GlaxoSmithKline in 3 clinical trials (2 phase I and 1 phase II) for psoriasis and ulcerative colitis. The ulcerative colitis phase II trial was terminated after an interim analysis [83], but phase I results show good tolerability, safety and inflammation regulation profiles [84].
- **HLX26**, developed by Fosun Pharma in 2 phase I clinical trials (NCT05078593 and NCT05400265), where its safety, tolerability, pharmacokinetic characteristics and preliminary efficacy are being evaluated alone and in combination with anti-PD-1 treatments in patients with solid tumors or lymphoma.
- **IBI110**, developed by Innovent Biologics in a phase I clinical trial alone and in combination with anti-PD-1 in patients with relapsed or refractory diffuse large B cell lymphoma (r/r DLBCL) (NCT05039658).
- **INCAGN02385**, is being developed by Incyte in 4 clinical trials (1 phase I, 1 phase I/II and 2 phase II) alone (NCT03538028) or in combination (NCT04370704, NCT05287113, NCT04586244) with anti-PD-1 and anti-TIM-3 immune checkpoint therapies. Preliminary data shows good tolerability profiles [85].
- **LAG525** or **IMP701** developed by Novartis in 5 clinical trials (1 phase I, 1 phase I/II and 3 phase II), alone or in combination with anti-PD-1 blockers. The structure of this antibody consists of 4 subunits, 16 disulfide bridges and 2 N-glycosylation sites, with an estimated molecular weight of 147 kDa [86]. Preliminary data demonstrate promising pharmacokinetics, antitumour activity and safety alone and in combination [87,88,89].
- **MK-4830** or **favezelimab**, developed by Merck in 8 clinical trials (1 phase I, 5 phase I/II, 1 phase II and 1 phase III), alone or in combination with anti-PD-1, oxaliplatin, Leucovorin (Calcium Folinate), Fluorouracil [5-FU] and lenvatinib, showing manageable safety and tolerability alone and in combination. In fact, anti-LAG-3/anti-PD-1 combination showed a 6.3% objective response rate, better than the monotherapy treatment, with similar treatment-related adverse effects [90,91]. The structure of this antibody consists of 4 subunits, 16 disulfide bridges and 2 glycosylation sites, with an estimated molecular weight of 146 kDa [92].
- **REGN3767** or **fianlimab**, developed by Regeneron Pharmaceuticals and Sanofi in 3 clinical trials (1 phase I, 1 phase II and 1 phase III), promotes T cell activation and T cell mediated cytotoxicity with good pharmacokinetics and toxicology profiles in vitro and in vivo [93]. The structure of the antibody is composed of 4 subunits, 16 sulfide bridges and 2 N-glycosilation sites [94]. Early efficacy and antitumor activity were also suggested in the preliminary clinical trials results. Its combination with cemiplimab also showed a good safety profile [95,96,97]. The combination with anti-PD-1, and cemiplimab is being evaluated in phase I (NCT03005782), II (NCT01042379) and III (NCT05352672) trials while it is being studied alone in the NCT03005782 phase I trial. Interestingly, anti-LAG-3 PET tracers (89Zr-DFO-REGN3767) are being clinically developed to establish the tracer biodistribution and dosimetry, monitoring the response to REGN3767 treatment (NCT05346276, NCT04706715, NCT04566978). However, these clinical trials are not being considered in this review as LAG-3 targeting clinical trials, because their main purpose is establishing PET scanning as a diagnostic method.
- **Sym022**, developed by Symphogen in 3 phase I clinical trials, is being evaluated for dose-escalation and dose-expansion alone (NCT03489369) or in combination (NCT03311412, NCT04641871) with anti-PD-1 and anti-TIM-3 immunotherapies. The treatment combination showed synergic antitumor activity in preclinical models [98,99].
- **TSR-033**, is being developed by Tesaro in 2 phase I clinical trials, alone and in combination with anti-PD-1 and anti-TIM-3 treatments (NCT03250832, NCT02817633). The combination with PD-1 blockers increases CD4 T cell activation and IL-2 production and cell proliferation [100]. Phase I preliminary data indicates good safety and tolerability.

#### 2.2.2. Anti-LAG-3 Bispecific Antibodies

- **ABL501** is being developed by ABL Bio in a phase I trial for the treatment of any progressive, locally advanced (unresectable) or metastatic solid tumor (NCT05101109). This bispecific antibody blocks PD-L1 and LAG-3 as a single agent. Dose-escalation analysis is being conducted. The dosing interval to be used in the dose-expansion part will be re-evaluated based on the emerging safety and pharmacokinetics data from the dose-escalation part of the study. It promotes enhanced human T cell activation in vitro and potentiates antitumor responses of T cells through DC activation [101,102].
- **IBI323,** a LAG-3/PD-L1 bispecific antibody, is being developed by Innovent Biologics in a phase I clinical trial alone and in combination with chemotherapy in patients with advanced malignancies. The purpose of this study is to evaluate IBI323 safety, tolerability and efficacy. It enhances tumor-specific immunity in vitro [103].
- **MGD013** or **Tebotelimab**, a LAG-3/PD-1 bispecific DART^®^ antibody, is being developed by MacroGenetics in 7 clinical trials (3 phase I, 1 phase I/II, 1 phase II and 2 phase II/III) in patients with unresectable or metastatic neoplasms (NCT03219268), patients with advanced or metastatic solid tumors who failed prior treatment (NCT04178460), melanoma (NCT04653038), liver cancer (NCT04212221), Head and Neck Cancer (NCT04634825, NCT04082364) and HER2+ Gastric/GEJ Cancer (NCT04082364), to evaluate its safety and efficacy, alone or in combination with margetuximab (anti-HER2), niraparib (a selective PARP1/2 inhibitor), Brivanib Alaninate (Multitargeted tyrosine kinase inhibitor) and enoblituzumab (Anti-B7-H3 antibody). Preliminary results showed good tolerability, safety and antitumour activity profiles [104].
- **RO7247669**, a LAG-3/PD-1 bispecific antibody, is being developed by Hoffmann-La Roche in 1 phase I and 1 phase II clinical trials in patients with advanced and/or metastatic solid tumors (NCT04140500) and advanced or metastatic squamous cell carcinoma of the oesophagus (NCT04785820), alone or in combination with a PD-1/TIM-3 bispecific antibody or an anti-PD-1 single agent.
- **XmAb^®^22841** or **pavunalimab**, a LAG-3/CTLA-4 bispecific antibody, is being developed by Xencor in a phase I clinical trial (NCT03849469), alone and in combination with anti-PD-1 as a single agent in selected advanced solid tumors. It enhances antitumor activity, T cell activation, cytokine secretion and cell proliferation [105].
- **EMB-02**, a LAG-3/PD-1 bispecific antibody, is being developed as a single treatment agent by EpimAb Biotherapeutics in a phase I/II clinical trial (NCT04618393) in advanced solid tumors. Dose escalation followed by cohort expansion will be performed. In vivo preclinical data showed antitumor activity in anti-PD-1 resistant models.
- **FS118**, a LAG-3/PD-L1 bispecific antibody, is being developed as a single agent treatment by F-star Therapeutics in a phase I/II clinical trial (NCT03440437) in patients with advanced malignancies, to determine dosing and toxicity. It enhanced T cell activation and antitumor activity in vitro and in vivo [105,106,107]. Preliminary clinical trial data showed good pharmacodynamics and tolerability profiles. [108,109].
- **CB213** Humabody^®^, a PD-1xLAG-3 antagonist developed by Crescendo Biologics Ltd., have recently entered a partnership with Cancer Research UK for its clinical development into a future phase I clinical trial ([110]). This bispecific molecule binds and blocks with high affinity PD1 and LAG-3 on PD-1+LAG-3+ T cells, induces ex vivo T cell proliferation of dysfunctional T cells from NSCLC patients, with superior activity than anti-PD-1 alone and suppress tumor growth in vivo [111].

#### 2.2.3. LAG-3 Fusion Proteins

Two different LAG-3 fusion proteins are being developed in several phase I (9), I/II (2), and II (4) trials: IMP321 or Eftilagimod Alpha or Efti (Immutep) and EOC202 (Taizhou EOC Pharma) (Table S3). EOC202 is a recombinant human LAG-3 fusion protein injection combined with albumin-bound paclitaxel for the treatment of patients with HR+, HER2- advanced breast cancer with progression after endocrine therapy (NCT05322720). On the other hand, IMP321 is the only soluble recombinant form of LAG-3 that is being clinically developed. In fact, IMP321 was the first LAG-3 targeted molecule to be studied in the clinic in 2006.

- **IMP321**, Eftilagimod Alpha or Efti, a LAG-3 soluble fusion protein, is being developed by Immutep in 14 clinical trials (9 phase I, 2 phase I/II and 3 phase II) for the treatment of advanced solid tumors, hepatitis B and flu. IMP321 is being developed as an adjuvant and immune modulator for cancer and vaccines against infectious diseases, as well as an anticancer treatment agent. It is being tested alone and in combination with chemotherapy (gemcitabine), anti-PD-L1, anti-PD-1, paclitaxel, hepatitis B antigen (without alum), a reference flu antigen, Melan-A VLP vaccine and melanoma tumor-specific peptides. Data shows that IMP321 enhances T cell activation and proliferation, humoral, effector and adaptive immunity, cytokine release, immunogenicity and antitumor activity, with good tolerability, efficacy and safety profiles [112,113,114,115,116,117,118,119,120].
- **EOC202**, a recombinant human LAG-3 fusion protein, is being developed by Taizhou EOC Pharma in a phase II clinical trial (NCT05322720) in HR+, HER2- advanced breast cancer with progression after endocrine therapy to evaluate the PFS for EOC202 combined with albumin-bound paclitaxel versus albumin-bound paclitaxel alone.

#### 2.2.4. Anti-LAG-3 CAR-T Cells

One phase I/II clinical trial (NCT05410717) is evaluating Claudin6 targeting CAR-NK cells for Stage IV Ovarian Cancer, refractory testis cancer and recurrent endometrial cancer (Table S4). To enhance the killing capability, some CAR-NK cells in this trial are genetically engineered to express and secret IL7/CCL19 and/or SCFVs against PD1/CTLA4/Lag3. The one study arm evaluates engineering Claudin6 targeting CAR combined with/or without IL7/CCL19 and/or scfv against PD1/CTLA4/Lag3 secreting vector into NK cells, which are isolated from patients with advanced ovarian cancer or other cancers with expression of Claudin6, and then transfusing them back the patients.

### 2.3. Opdualag and Its Pathway towards the Clinic

On 18 March2022, the FDA approved Opdualag as a first line treatment for unresectable or metastatic melanoma at a fixed dose combination. This approval signified a major historical achievement for Bristol-Myers Squibb, and a remarkable milestone for the landscape of cancer treatments. This therapeutic strategy established for the first time a next-generation LAG-3 blocker for clinical use. Opdualag consists of a pre-mixed combination of two IgG4 kappa monoclonal antibodies, nivolumab 480 mg (anti-PD-1,146 kDa) and relatlimab (BMS-98621) 160 mg (anti-LAG-3, 148 kDa) both expressed in Chinese Hamster Ovary (CHO) cell lines. This combination is prepared and provided to the patient through intravenous (IV) infusions every 4 weeks until disease progression or unacceptable toxicity occurs [121]. Its list price is $13,694.27, and it is indicated for adults and paediatric patients over the age of 12 with unresectable or metastatic melanoma that has spread or cannot be removed by surgery. 

Nivolumab (opdivo) is an anti-PD-1 monoclonal antibody from Bristol-Myers Squibb clinically developed in more than 35,000 patients including Phase 3, for the treatment of a variety of tumor types. Currently approved in more than 65 countries (including the United States, the European Union, Japan and China) Opdivo was the first anti-PD-1 immune checkpoint blocker to be approved for clinical use in July 2014. Later in 2015, the combination of nivolumab with ipilimumab (Yerboy), a CTLA-4 blocking monoclonal antibody, was approved for metastatic melanoma, demonstrating to be safe and effective. In addition, preclinical and clinical studies showed that nivolumab and relatlimab combination reactivated T and NK cell-mediated responses, enhanced T cell activation and cytokine production, restoring the effector functions of exhausted T cells [82].

The FDA Oncology Center of Excellent conducted a Project Orbis review on the novel drug in collaboration with the Australian Therapeutic Goods Administration (TGA), and Switzerland’s Swissmedic to provide a review of the oncology drugs framework among international partners. This was carried out using the Real-Time Oncology Review (RTOR) pilot program to streamline data submission prior to the clinical application filing and the Assessment Aid (an applicant voluntary submission). The application by Bristol-Myers Squibb (sponsor, study director, and responsible party of the trial) was then granted priority review, fast track, and orphan drug designation [122]. 

Opdualag was clinically evaluated in RELATIVITY-047 (NCT03470922), an interventional multi-institutional (127 locations all over the glove), randomized (1:1), parallel assignment interventionist, quadruple-blinded (participant, care provider, investigator, outcomes assessor) phase II/III trial that enrolled 714 patients (>12 years of age). The actual study start date was 11 April 2018, the primary completion date was 25 January 2021 and the estimated study completion date is 30 November 2023. The purpose of this study is to determine whether relatlimab in combination with nivolumab is more effective than nivolumab as a monotherapy in treating unresectable melanoma or metastatic melanoma. 

Inclusion criteria were histologically confirmed Stage III (unresectable) or Stage IV melanoma per the AJCC staging system, not having had prior systemic anticancer therapy for these cancers, and that tumor tissue from an unresectable or metastatic site of disease must be provided for biomarker analyses. The two sexes were eligible for the study. Participants must have a documented BRAF status prior to randomization. Exclusion criteria were that participants must not have active brain metastases or leptomeningeal metastases, uveal melanoma, nor active, known, or suspected autoimmune disease. Healthy volunteers were not accepted. No lifestyle restrictions were required during treatment.

The primary outcome in the trial was PFS determined by Blinded Independent Central Review (BICR) using RECIST v1.1 from randomization to date of first documented tumor progression or death (up to approximately 33 months). The secondary outcome were Overall Survival (OS) and Overall Response Rate (ORR), from randomization to the date of death (up to approximately 3 years). Other outcome measures were the number of participants experiencing adverse events (AEs), serious adverse events (SAEs), AEs leading to discontinuation, laboratory abnormalities in specific liver and thyroid tests from first dose to 30 days after last dose of the study (up to approximately 33 months), as well as the number deaths from first dose up to approximately 33 months. 

Patients received IV treatment (nivolumab 480 mg and relatlimab 160 mg) every 4 weeks (*n* = 355) or nivolumab 480 mg by IV infusion every 4 weeks (*n* = 359) (clinical protocol CA224047) [123]). The median treatment duration was 6 months (0–31 months range) in Opdualag-treated patients and 5 months (0–32 months range) in nivolumab-treated patients [124]. To compare the side effect profile of PD-1 monoblockade (Nivolumab arm) *versus* dual therapy (Opdualag arm), the most common (>10%) AEs and SAEs (>1%) are presented in Table 1. Briefly, opdualag potentially breaks peripheral tolerance and induces immune-mediated adverse reactions (IMARs). It can cause serious side effects. Indeed, 0.8% of the Opdualag-treated patients (3 patients) presented fatal AEs, 18% of the participants discontinued from Opdualag treatment due to AEs, and 43% of them had AEs that required dosage interruptions. In addition, the treatment can cause reproductive toxicity to pregnant women. No safety or effectiveness differences were observed upon the age of patients.

Opdualag demonstrated a statistically significant improvement in PFS when compared to nivolumab alone (HR = 0.75; 95% [CI]: 0.62, 0.92; *p*-value = 0.0055) with a PFS median of 10.1 months for the Opdualag arm (95% CI: 6.4, 15.7) versus 4.6 months in the nivolumab arm (95% CI: 6.4, 15.7). Opdualag did not showed a significant improvement in OS when compared to nivolumab alone (HR = 0.80; 95% CI: 0.64, 1.01) with a not reached OS median for Opdualag arm (95% CI: 34.2, NR) versus 34.1 months in the nivolumab arm (95% CI: 25.2, NR) [122,125,126,127].

## 3. Behind the Steps of LAG-3 towards the Clinic

The rationale behind the blockade of multiple immune checkpoint receptors relies on their varied mechanisms of action, whose combination may converge to a synergistic effect. Here, three main mechanistic pathways are addressed [128,129]:

1. Immune activation at lymph nodes and peripheral tissues. The activation of T cells requires not only the antigen presentation through the MHC complex, but also a second costimulatory signal provided by APCs. T cells are initially primed at lymph nodes, although the interaction with other immune cell populations in peripheral tissues as well as in the tumor microenvironment may also provide immunoregulatory signals. These signals mediate the acquisition of effector functions or immunosuppressive phenotypes by T cells depending on the engagement of costimulatory or checkpoint receptors. In this case, immune checkpoint blockade would enhance T cell activation by APCs.
2. Priming of immune tolerogenic phenotypes. Some immune checkpoint receptors induce tolerogenic phenotypes on antigen-presenting cells (APCs) and prime Tregs.
3. Induction of T-cell dysfunction. Sustained antigen presentation and stimulation with inflammatory cytokines induce T cell exhaustion, characterized by reduced proliferation and effector functions. However, cytotoxic functions may be rescued by immune checkpoint receptors blockade with monoclonal antibodies. It has been reported that exhausted T cells show increased expression of multiple checkpoint receptors, which may interfere with the response rate of patients to ICB monotherapies.

Whereas the blockade of the PD-1/PD-L1 axis has focused attention on the ICB field due to its impressive and durable clinical responses, a plethora of immune checkpoint receptors have been identified in the last years, which are gaining attention. Apart from the classical checkpoints CTLA-4, PD-1, and PD-L1, the so-called second generation of ICIs targeting alternative receptors are gaining increasing relevance in their pathway toward their clinical application [130]. Some years ago, LAG-3 was considered one of these promising ‘next generation’ checkpoints. Importantly, after the approval of Opdualag by the FDA for its clinical use, this checkpoint has jumped to the first line of immunotherapeutic agents. Thus, LAG-3 has paved the way for other checkpoints. We review below the pre-clinical degree of development of the main ‘second generation’ checkpoint inhibitors that may follow the steps of LAG-3 in a near future.

### 3.1. TIM-3

TIM-3 (T-cell immunoglobulin and mucin domain-containing protein 3) is expressed by several cell populations, including CD4 and CD8 T cells [131], Tregs [132], myeloid cells [133], and NKs [134]. Its known ligands are galectin-9 [135], CEACAM-1 [136], phosphatidylserine [137], and HMGB1 [138], which interact with different regions of the TIM-3 extracellular immunoglobulin V domain, leading to the activation of differential signaling cascades. TIM-3 engagement would be mediated by differential expression of each ligand in each tissue microenvironment. In the context of cancer, TIM-3 expression has been found to be a marker of CD8 tumor-infiltrating T lymphocytes (TILs) dysfunctionality and Treg expansion, and correlates with tumor progression [139]. Consequently, the TIM-3 blockade restores antitumor CD8 T cell responses [140] and mediates Treg depletion [141]. Importantly, TIM-3 and PD-1 are usually co-expressed both in CD4 and CD8 T cells, being these cells highly dysfunctional in terms of impaired production of effector IFNγ, TNFα, and IL-2, thus promoting effector T cells exhaustion [140]. Consistently, a co-blockade of PD-1 and TIM-3 leads to a stronger reinforcement of T cell responses compared to PD-1 monoblockade, as shown in preclinical models [142,143,144]. TIM-3 as a single immunotherapy has shown limited results in clinical trials. Nevertheless, synergistic effects are boosted by the combination of anti-TIM-3 monoclonal antibodies with other ICIs, such as CTLA-4 and PD-1. Moreover, opposing these classical checkpoints, TIM-3 expression is restricted to terminally differentiated T cells and intratumoral T regs, thus avoiding broad AEs elicited by CTLA-4 [145] and PD-1 blockade [146,147].

Many ongoing clinical trials aim to analyze the co-blockade of TIM-3 and PD-1 (Table S5). Most of the therapies designed to target TIM-3 are fully humanized IgG4 or IgG1 blocking antibodies that bind with high affinity to TIM-3. Moreover, several bi-specific antibodies for the dual blockade of PD-1 and TIM-3 have also been developed. However, only the anti-TIM-3 antibody cobolimab (TSR-022) has progressed to a phase II clinical trial up to date. TSR-022 is a humanized IgG4 monoclonal antibody against TIM-3, developed by Tesaro and GlaxoSmithKline. Early results from preclinical studies revealed that cobolimab enhanced IL-2 production by CD4 T cells in MLR assays in vitro, while in vivo studies demonstrated a good tolerability profile. Altogether, these results encouraged its evaluation in clinical assays [147,148]. Following preliminary efficacy results from the phase I AMBER study (NCT02817633) [149], the combination of cobolimab with the anti-PD-1 inhibitor dostarlimab (TSR-042) showed preliminary anti-tumor activity with acceptable tolerability in a range of advanced solid tumors including NSCLC, melanoma and peritoneal mesothelioma. The second phase of the study will evaluate the safety and efficacy of the combination of cobolimab with the anti-PD-1 blocking antibody dostarlimab in disease-specific cohorts including patients with NSCLC who received previous PD-L1 blockade immunotherapy and colorectal cancer patients with previous treatments.

### 3.2. TIGIT

TIGIT (T cell immunoreceptor with Ig and ITIM domains) is a member of the PVR-nectin family. It is expressed on CD4, CD8, γδ T cells, Treg and NKs. Its main ligand is PVR, which is usually overexpressed on tumor cells and tumor-associated myeloid cells [150,151], although other proteins, such as CD112 and CD113, also interact with this receptor. Its ligation suppresses T cell activation [152,153]. The mechanism of action of the TIGIT-PVR axis differs from the classical immune checkpoints, thus making it an attractive target for immunotherapy. Unlike PD-1 and CTLA-4, NKs constitutively express TIGIT, and its expression on intratumoral NKs is upregulated [154]. Therefore, anti-TIGIT treatments show the particular ability to target at the same time the two main antitumor effector populations, T lymphocytes, and NKs. Moreover, TIGIT blockade abrogates MDSCs-mediated NK suppression [155,156] and immunosuppressive activity of Tregs displaying a high TIGIT expression [157]. Since this checkpoint receptor establishes an immunosuppressive tumor microenvironment, its overexpression correlates with poor clinical outcomes [158,159,160,161]. Preclinical data in murine models demonstrate that TIGIT blockade resulted in reduced tumor growth and prolonged survival rates [154,162]. However, the co-blockade of TIGIT and PD-1/PD-L1 is required to achieve clinical responses in a therapeutic setting [152,154,163,164].

Several dozens of anti-TIGIT blocking antibodies or bispecific antibodies against PD-1/TIGIT are currently undergoing clinical trials in diverse stages of development (Table S6). Most of them are IgG1 isotype antibodies that interact with Fcγ receptors. Among them, the most promising candidates to reach the market up to date are ociperlimab, tiragolumab, and vibostolimab, all of them under evaluation in phase III clinical trials.

Ociperlimab is a fully humanized IgG1 antibody developed by BeiGene that binds TIGIT with high affinity and specificity thanks to its intact IgG Fc function [165]. The combination of ociperlimab plus the anti-PD-1 antibody tislelizumab (BGB-A317) is currently under evaluation in patients diagnosed with distinct advanced or metastatic tumors, including NSCLC, esophageal squamous cell carcinoma, and cervical cancer. Preliminary results showed that this combination was well tolerated, showed antitumor activity, and elicited AEs consistent with tislelizumab monotherapy [166]. The phase III clinical trial AdvanTIG-302 (NCT04746924), sponsored by the pharmaceutical company BeiGene, is a multicentre, international, randomized, and double-blinded study analyzing the combo ociperlimab plus tislelizumab for the first-line treatment of patients with locally advanced, unresectable, or metastatic NSCLC whose tumors exhibit high PD-L1 expression and do not harbor EGFR-sensitizing mutations or ALK translocations.

Tiragolumab is a fully humanized IgG1 antibody developed by Hoffmann-La Roche and Genentech. It is the most advanced anti-TIGIT therapy, currently in late-stage clinical development. The combination of tiragolumab with the anti-PD-L1 antibody atezolizumab (Tecentriq^®^) was evaluated in the phase II clinical trial CITYSCAPE [167], whose preliminary results showed an 18% higher ORR compared to atezolizumab alone treated group, while the risk of death was reduced by 38%. Based on these positive data, tiragolumab received breakthrough therapy designation by FDA [168] for first-line treatment of NSCLC patients with high tumor PD-L1 expression and no EGFR or ALK mutations, thus making it an excellent candidate to reach the market in a near future. The ongoing phase III clinical trial SKYSCRAPER (NCT04294810) is currently evaluating the combo tiragolumab plus atezolizumab. Recent results indicated that PFS end-points of the combination arm with chemotherapy were not reached [169], while the study continues until mature OS data from the rest of the trial arms will be available. By the way, the treatment has been shown to be well tolerated, and no new safety signals were identified.

Vibostolimab is a humanized anti-TIGIT IgG1 antibody developed by Merck. Applied as a monotherapy or in combination with the anti-PD-1 antibody pembrolizumab, it showed a manageable safety profile and promising therapeutic potential in first-line treatment patients [170]. However, only modest antitumor activity was achieved in NSCLC patients refractory to anti-PD-1/PD-L1 immunotherapy. The coformulation of pembrolizumab/vibostolimab is currently being evaluated in the phase III clinical trial KEYVIBE (NCT04738487) in PD-L1 positive metastatic NSCLC as first-line treatment compared to pembrolizumab alone [171].

### 3.3. CD137

CD137 belongs to the TNF receptor superfamily (TNFRSF). It is expressed on CD4 and CD8 T cells shortly after antigen exposure [172], and its ligation, together with the TCR, delivers co-stimulatory signals that promote T cell expansion with memory phenotypes and stronger effector capacities in terms of cytotoxic capacities and cytokine production [173]. Interestingly, these effects are particularly pronounced on CD8 T cells rather than on CD4 lymphocytes [173]. Apart from CD137, other costimulatory receptors of the TNFRSF family, such as OX40, GITR, or CD27, have been targeted with agonistic antibodies to promote anti-tumor T cell responses. However, most of them have shown weak or not durable responses according to preliminary results in phase I clinical trials when administered as monotherapies [174,175,176,177,178,179,180,181,182,183,184,185,186,187]. Apart from toxicity-related concerns, the determination of optimal dosing schedules seems to be of great importance, since a minimum half-life is required for T cell engagement, while persistent stimulation may lead to T cell exhaustion. CD137 appears to be the most robust target among the TNFRSF costimulatory receptors under current clinical investigation. Two main agonistic antibodies have been developed up to date, urelumab, and utomilumab.

Urelumab (BMS-663513) is a humanized IgG4 agonistic antibody to CD137 developed by Bristol-Myers Squibb. The first results from clinical trials have demonstrated clinical responses in patients with advanced solid tumors and lymphomas [188,189]. However, hepatotoxicity concerns arose associated with required doses above the maximum tolerated limits [190]. Utomilumab (PF-05082566) is a humanized IgG2 agonistic antibody to CD137 developed by Pfizer, that has been proved to be well tolerated in a phase I clinical trial (NCT01307267) [191], although limited efficacy was achieved. Due to security profiles and efficacy limitation issues, the combination of CD137 agonistic antibodies with other immuno oncology agents is being analyzed in order to achieve sufficient therapeutic effects [192,193,194]. Bispecific antibodies for the simultaneous targeting of CD137 and tumour-associated antigens (TAA) are attracting increasing attention as a strategy to reduce off-target toxicities (Table S7).

### 3.4. ICOS

Inducible T cell costimulatory (ICOS) is an activating costimulatory checkpoint expressed on activated T cells. Its unique ligand, ICOSL, is expressed by APCs and somatic cells, including tumor cells [195,196,197,198]. The ICOS/ICOSL pathway represents an attractive target for immunotherapy, due to its dual role in the context of cancer. While its ligation promotes an increase in the Teff/Treg ratio in the tumor microenvironment and differentiation of Th1 TILs [199,200], its sustained activation induces immunosuppression mediated by Tregs [201,202,203,204], thus displaying both pro- and anti-tumorigenic potential. Indeed, both agonistic and blocking antibodies that target this pathway are being evaluated in clinical trials (Table S8).

Preliminary results from phase I clinical trials show that agonistic monoclonal antibodies targeting ICOS rendered promising clinical responses with good safety and tolerability profiles, whereas ICOS antagonistic antibodies, aimed at ICOS expressing-Tregs abrogation, did not achieve remarkable response rates [205,206,207,208]. Interestingly, preclinical results demonstrated a strong synergy between ICOS antibodies and other ICB agents, such as CTLA-4 or PD-1/PD-L1 [209]. Therefore, most ongoing clinical trials are evaluating combinations of anti-ICOS agonistic and antagonistic antibodies with conventional ICB therapies.

Vopratelimab (JTX-2011) is the most advanced ICOS-agonistic antibody up to date. It is an IgG1 monoclonal antibody developed by Jounce Therapeutics. The first-in-human phase I/II ICONIC trial (NCT0290422) revealed that this antibody boosts the activation and proliferation of primed CD4 T effector cells and leads to improved clinical outcomes in patients treated with this antibody both as a monotherapy and in combination with the anti-PD-1 inhibitor nivolumab [210,211]. Following promising results from this phase I clinical trial with no adverse safety and tolerability concerns, it is currently being analyzed in the SELECT phase II trial (NCT04549025) in combination with another PD-1 inhibitor (pimivalimab, JTX-4014) in NSCLC patients [212].

## 4. Conclusions

ICIs have revolutionized oncology medical practice since the FDA approval of the first ICI 11 years ago. However, one of the main current challenges in oncology is that many patients do not respond to treatment due to intrinsic and extrinsic factors, which is a major clinical problem. In light of this, LAG-3 is one of the most important next-generation immune checkpoint molecules, playing a similar role to PD-1 and CTLA-4 inhibitory molecules. LAG-3 plays a critical role in regulating T cell homeostasis, acting as an inhibitor of T cell activation, proliferation, cytokine secretion, and effector functions. Thus, 108 interventional clinical trials are evaluating 19 different LAG-3 targeting molecules in phases I, I/II, II, II/III, and III, demonstrating strong positive results, including promising bispecific molecules targeting LAG-3 with other immune checkpoint inhibitors, especially with anti-PD-1 blockers.

The recent approval of Opdualag, a novel immune checkpoint inhibitor fixed-dose treatment combination of nivolumab (anti-PD-1) and relatlimab (anti-LAG-3) developed by Bristol Myers Squibb for the treatment of first-line unresectable melanoma or melanoma that has spread (advanced melanoma) is a significant large milestone in the clinical landscape of cancer treatment. This LAG-3 and PD-1 co-blockade has proven to be safe and effective, providing greater benefit in terms of PFS when compared to PD-1 blockade alone, with manageable toxicity profiles. Remarkably, it more than doubles the median PFS when compared to nivolumab monotherapy (10.1 months versus 4.6 months), demonstrating to be safe and effective. This regulatory approval opens a door to the entry of more promising LAG-3 targeting molecules in the clinical practice market, and to an extension of Opdualag indications to other malignancies, supporting the accumulating evidence that indicates that LAG-3 will play a pivotal role in cancer treatment equivalent to current anti-PD-1/anti-PD-L1 treatments.

## Figures and Tables

**Table 1:** Summary of the most common AEs and SAEs associated with the treatment with Opdualag compared to Nivolumab alone in the RELATIVITY-047 clinical trial (NCT03470922).

		Opdualag (*n* = 355)	Nivolumab (*n* = 359)
**AEs**	Musculoskeletal pain	54.6%	33.1%
	Fatigue	29.3%	20.6%
	Asthenia	13.5%	9.2%
	Pyrexia	12.4%	9.2%
	Headache	18.0%	13.1%
	Cough	14.1%	10.6%
	Rash	17.4%	13.6%
	Pruritus	24.8%	17.3%
	Diarrhea	23.1%	17.3%
	Nausea	17.7%	16.4%
	Constipation	11.0%	7.0%
	Decreased appetite	15.5%	7.5%
	Anaemia	14.1%	10.3%
	Increased AST	9.9%	4.7%
	Increased ALT	10.1%	5.85%
	Hypothyroidism	16.3%	13.1%
**SAEs**	Anaemia	1.4%	1.1%
	Acute myocardial infarction	1.1%	0.6%
	Myocarditis	1.1%	0.3%
	Adrenal insufficiency	1.4%	0.0%
	Colitis	1.4%	0.3%
	Diarrhea	1.1%	0.8%
	General health deterioration	0.6%	1.7%
	Pyrexia	0.9%	1.4 %
	Pneumonia	1.4%	0.8%
	Urinary tract infection	0.9%	1.7%
	Back pain	1.1%	0.6%
	Malignant neoplasm progression	11.0%	13.1%
	Metastases to central nervous system	1.1%	0.8%
	Pneumonitis	1.1%	0.3%

## Data Availability

Not applicable.

## Supplementary Materials

The following supporting information can be downloaded at: https://www.mdpi.com/article/10.3390/cells11152351/s1, Table S1: Ongoing clinical trials involving anti-LAG-3 monoclonal antibodies (https://clinicaltrials.gov/, accessed on June 2022); Table S2: Ongoing clinical trials involving anti-LAG-3 bispecific antibodies antibodies (https://clinicaltrials.gov/, accessed on June 2022); Table S3: Ongoing clinical trials involving LAG-3 fusion proteins antibodies (https://clinicaltrials.gov/, accessed on June 2022); Table S4: Ongoing clinical trials involving anti-LAG-3 CAR-T cell therapies (https://clinicaltrials.gov/, accessed on June 2022); Table S5: Ongoing clinical trials involving anti-TIM-3 blocking antibodies (https://clinicaltrials.gov/, accessed on June 2022); Table S6: Ongoing clinical trials involving anti-TIGIT blocking antibodies (https://clinicaltrials.gov/, accessed on 29 June 2022); Table S7: Ongoing clinical trials involving anti-CD137 bispecific antibodies (https://clinicaltrials.gov/, accessed on 29 June 2022); Table S8: Ongoing clinical trials involving anti-ICOS monoclonal antibodies (https://clinicaltrials.gov/, accessed on June 2022).

## References

[1] De Erauso L.C., Zuazo M., Arasanz H., Bocanegra A., Hernandez C., Fernandez G., Garcia-Granda M.J., Blanco E., Vera R., Kochan G. (2020). Resistance to PD-L1/PD-1 Blockade Immunotherapy. A Tumor-Intrinsic or Tumor-Extrinsic Phenomenon?. Front. Pharmacol..

[2] Chocarro L., Blanco E., Arasanz H., Fernández-Rubio L., Bocanegra A., Echaide M., Garnica M., Ramos P., Fernández-Hinojal G., Vera R. (2022). Clinical landscape of LAG-3-targeted therapy. Immunol.-Oncol. Technol..

[3] Chocarro L., Blanco E., Zuazo M., Arasanz H., Bocanegra A., Fernández-Rubio L., Morente P., Fernández-Hinojal G., Echaide M., Garnica M. (2021). Understanding LAG-3 Signaling. Int. J. Mol. Sci..

[4] Triebel F., Jitsukawa S., Baixeras E., Roman-Roman S., Genevee C., Viegas-Pequignot E., Hercend T. (1990). LAG-3, a novel lymphocyte activation gene closely related to CD4. J. Exp. Med..

[5] Huard B., Prigent P., Pagès F., Bruniquel D., Triebel F. (1996). T cell major histocompatibility complex class II molecules down-regulate CD4+ T cell clone responses following LAG-3 binding. Eur. J. Immunol..

[6] Huard B., Tournier M., Hercend T., Triebel F., Faure F. (1994). Lymphocyte-activation gene 3/major histocompatibility complex class II interaction modulates the antigenic response of CD4+ T lymphocytes. Eur. J. Immunol..

[7] Andrews L.P., Marciscano A.E., Drake C.G., Vignali D.A.A. (2017). LAG3 (CD223) as a cancer immunotherapy target. Immunol. Rev..

[8] Workman C.J., Cauley L.S., Kim I.-J., Blackman M.A., Woodland D.L., Vignali D.A.A. (2004). Lymphocyte Activation Gene-3 (CD223) Regulates the Size of the Expanding T Cell Population Following Antigen Activation In Vivo. J. Immunol..

[9] Workman C.J., Dugger K.J., Vignali D.A.A. (2002). Cutting Edge: Molecular Analysis of the Negative Regulatory Function of Lymphocyte Activation Gene-3. J. Immunol..

[10] Williams J.B., Horton B.L., Zheng Y., Duan Y., Powell J.D., Gajewski T.F. (2017). The EGR2 targets LAG-3 and 4-1BB describe and regulate dysfunctional antigen-specific CD8+ T cells in the tumor microenvironment. J. Exp. Med..

[11] Huang R.-Y., Eppolito C., Lele S., Shrikant P., Matsuzaki J., Odunsi K. (2015). LAG3 and PD1 co-inhibitory molecules collaborate to limit CD8+ T cell signaling and dampen antitumor immunity in a murine ovarian cancer model. Oncotarget.

[12] Grosso J.F., Goldberg M.V., Getnet D., Bruno T.C., Yen H.-R., Pyle K.J., Hipkiss E., Vignali D.A.A., Pardoll D.M., Drake C.G. (2009). Functionally Distinct LAG-3 and PD-1 Subsets on Activated and Chronically Stimulated CD8 T Cells. J. Immunol..

[13] Grosso J.F., Kelleher C.C., Harris T.J., Maris C.H., Hipkiss E.L., De Marzo A., Anders R., Netto G., Getnet D., Bruno T.C. (2007). LAG-3 regulates CD8+ T cell accumulation and effector function in murine self- and tumor-tolerance systems. J. Clin. Investig..

[14] Chihara N., Madi A., Kondo T., Zhang H., Acharya N., Singer M., Nyman J., Marjanovic N.D., Kowalczyk M.S., Wang C. (2018). Induction and transcriptional regulation of the co-inhibitory gene module in T cells. Nature.

[15] Blackburn S.D., Shin H., Haining W.N., Zou T., Workman C.J., Polley A., Betts M.R., Freeman G.J., A A Vignali D., Wherry E.J. (2008). Coregulation of CD8+ T cell exhaustion by multiple inhibitory receptors during chronic viral infection. Nat. Immunol..

[16] Camisaschi C., Casati C., Rini F., Perego M., De Filippo A., Triebel F., Parmiani G., Belli F., Rivoltini L., Castelli C. (2010). LAG-3 Expression Defines a Subset of CD4+CD25highFoxp3+ Regulatory T Cells That Are Expanded at Tumor Sites. J. Immunol..

[17] Workman C.J., Vignali D.A.A. (2005). Negative Regulation of T Cell Homeostasis by Lymphocyte Activation Gene-3 (CD223). J. Immunol..

[18] White A.M., Wraith D.C. (2016). Tr1-Like T Cells—An Enigmatic Regulatory T Cell Lineage. Front. Immunol..

[19] Huard B., Tournier M., Triebel F. (1998). LAG-3 does not define a specific mode of natural killing in human. Immunol. Lett..

[20] Baixeras E., Huard B., Miossec C., Jitsukawa S., Martin M., Hercend T., Auffray C., Triebel F., Piatier-Tonneau D. (1992). Characterization of the lymphocyte activation gene 3-encoded protein. A new ligand for human leukocyte antigen class II antigens. J. Exp. Med..

[21] Huang C.-T., Workman C.J., Flies D., Pan X., Marson A.L., Zhou G., Hipkiss E.L., Ravi S., Kowalski J., Levitsky H.I. (2004). Role of LAG-3 in Regulatory T Cells. Immunity.

[22] Zuazo M., Arasanz H., Fernández-Hinojal G., García-Granda M.J., Gato M., Bocanegra A., Martínez M., Hernández B., Teijeira L., Morilla I. (2019). Functional systemic CD 4 immunity is required for clinical responses to PD -L1/PD -1 blockade therapy. EMBO Mol. Med..

[23] Matsuzaki J., Gnjatic S., Mhawech-Fauceglia P., Beck A., Miller A., Tsuji T., Eppolito C., Qian F., Lele S., Shrikant P. (2010). Tumor-infiltrating NY-ESO-1–specific CD8 + T cells are negatively regulated by LAG-3 and PD-1 in human ovarian cancer. Proc. Natl. Acad. Sci. USA.

[24] Bruniquel D., Borie N., Hannier S., Triebel F. (1998). Regulation of expression of the human lymphocyte activation gene-3 (LAG-3) molecule, a ligand for MHC class II. Immunogenetics.

[25] Annunziato F., Manetti R., Cosmi L., Galli G., Heusser C.H., Romagnani S., Maggi E. (1997). Opposite role for interleukin-4 and interferon-γ on CD30 and lymphocyte activation gene-3 (LAG-3) expression by activated naive T cells. Eur. J. Immunol..

[26] Annunziato F., Manetti R., Tomasévic I., Giudizi M., Biagiotti R., Giannò V., Germano P., Mavilia C., Maggi E., Romagnani S. (1996). Expression and release of LAG-3-encoded protein by human CD4 + T cells are associated with IFN-γ production. FASEB J..

[27] Bae J., Lee S.J., Park C.-G., Lee Y.S., Chun T. (2014). Trafficking of LAG-3 to the Surface on Activated T Cells via Its Cytoplasmic Domain and Protein Kinase C Signaling. J. Immunol..

[28] Mao X., Ou M.T., Karuppagounder S.S., Kam T.-I., Yin X., Xiong Y., Ge P., Umanah G.E., Brahmachari S., Shin J.-H. (2016). Pathological α-synuclein transmission initiated by binding lymphocyte-activation gene 3. Science.

[29] Angelopoulou E., Paudel Y.N., Villa C., Shaikh M.F., Piperi C. (2020). Lymphocyte-Activation Gene 3 (LAG3) Protein as a Possible Therapeutic Target for Parkinson’s Disease: Molecular Mechanisms Connecting Neuroinflammation to α-Synuclein Spreading Pathology. Biology.

[30] Guo W., Zhou M., Qiu J., Lin Y., Chen X., Huang S., Mo M., Liu H., Peng G., Zhu X. (2019). Association of LAG3 genetic variation with an increased risk of PD in Chinese female population. J. NeuroInflamm..

[31] Lino A.C., Dang V.D., Lampropoulou V., Welle A., Joedicke J., Pohar J., Simon Q., Thalmensi J., Baures A., Flühler V. (2018). LAG-3 Inhibitory Receptor Expression Identifies Immunosuppressive Natural Regulatory Plasma Cells. Immunity.

[32] Kisielow M., Kisielow J., Capoferri-Sollami G., Karjalainen K. (2005). Expression of lymphocyte activation gene 3 (LAG-3) on B cells is induced by T cells. Eur. J. Immunol..

[33] Donia M., Andersen R., Kjeldsen J.W., Fagone P., Munir S., Nicoletti F., Andersen M.H., Straten P.T., Svane I.M. (2015). Aberrant Expression of MHC Class II in Melanoma Attracts Inflammatory Tumor-Specific CD4+ T- Cells, Which Dampen CD8+ T-cell Antitumor Reactivity. Cancer Res..

[34] Hemon P., Jean-Louis F., Ramgolam K., Brignone C., Viguier M., Bachelez H., Triebel F., Charron D., Aoudjit F., Al-Daccak R. (2011). MHC Class II Engagement by Its Ligand LAG-3 (CD223) Contributes to Melanoma Resistance to Apoptosis. J. Immunol..

[35] Huard B., Prigent P., Tournier M., Bruniquel D., Triebel F. (1995). CD4/major histocompatibility complex class II interaction analyzed with CD4- and lymphocyte activation gene-3 (LAG-3)-Ig fusion proteins. Eur. J. Immunol..

[36] Long L., Zhang X., Chen F., Pan Q., Phiphatwatchara P., Zeng Y., Chen H. (2018). The promising immune checkpoint LAG-3: From tumor microenvironment to cancer immunotherapy. Genes Cancer.

[37] Kouo T., Huang L., Pucsek A.B., Cao M., Solt S., Armstrong T., Jaffee E. (2015). Galectin-3 Shapes Antitumor Immune Responses by Suppressing CD8+ T Cells via LAG-3 and Inhibiting Expansion of Plasmacytoid Dendritic Cells. Cancer Immunol. Res..

[38] Li M., Feng Y., Fang S. (2017). Overexpression of ezrin and galectin-3 as predictors of poor prognosis of cervical cancer. Braz. J. Med. Biol. Res..

[39] Lu W., Wang J., Yang G., Yu N., Huang Z., Xu H., Li J., Qiu J., Zeng X., Chen S. (2017). Posttranscriptional regulation of Galectin-3 by miR-128 contributes to colorectal cancer progression. Oncotarget.

[40] Chung L.-Y., Tang S.-J., Wu Y.-C., Sun G.-H., Liu H.-Y., Sun K.-H. (2014). Galectin-3 augments tumor initiating property and tumorigenicity of lung cancer through interaction with β-catenin. Oncotarget.

[41] Ming Q., Celias D.P., Wu C., Cole A.R., Singh S., Mason C., Dong S., Tran T.H., Amarasinghe G.K., Ruffell B. (2022). LAG3 ectodomain structure reveals functional interfaces for ligand and antibody recognition. Nat. Immunol..

[42] Huard B., Mastrangeli R., Prigent P., Bruniquel D., Donini S., El-Tayar N., Maigret B., Dréano M., Triebel F. (1997). Characterization of the major histocompatibility complex class II binding site on LAG-3 protein. Proc. Natl. Acad. Sci. USA.

[43] Wang J., Sanmamed M.F., Datar I., Su T.T., Ji L., Sun J., Chen L., Chen Y., Zhu G., Yin W. (2019). Fibrinogen-like Protein 1 Is a Major Immune Inhibitory Ligand of LAG-3. Cell.

[44] Xu F., Liu J., Liu D., Liu B., Wang M., Hu Z., Du X., Tang L., He F. (2014). LSECtin Expressed on Melanoma Cells Promotes Tumor Progression by Inhibiting Antitumor T-cell Responses. Cancer Res..

[45] Zuazo M., Arasanz H., Bocanegra A., Chocarro L., Vera R., Escors D., Kagamu H., Kochan G. (2020). Systemic CD4 immunity: A powerful clinical biomarker for PD-L1/PD-1 immunotherapy. EMBO Mol. Med..

[46] Hernandez C., Arasanz H., Chocarro L., Bocanegra A., Zuazo M., Fernandez-Hinojal G., Blanco E., Vera R., Escors D., Kochan G. (2020). Systemic Blood Immune Cell Populations as Biomarkers for the Outcome of Immune Checkpoint Inhibitor Therapies. Int. J. Mol. Sci..

[47] Zuazo M., Arasanz H., Bocanegra A., Fernandez G., Chocarro L., Vera R., Kochan G., Escors D. (2020). Systemic CD4 Immunity as a Key Contributor to PD-L1/PD-1 Blockade Immunotherapy Efficacy. Front. Immunol..

[48] Zhang X., Zhao H., Shi X., Jia X., Yang Y. (2020). Identification and validation of an immune-related gene signature predictive of overall survival in colon cancer. Aging.

[49] Saka D., Gökalp M., Piyade B., Cevik N.C., Arik Sever E., Unutmaz D., Ceyhan G.O., Demir I.E., Asimgil H. (2020). Mechanisms of T-Cell Exhaustion in Pancreatic Cancer. Cancers.

[50] Deng W.-W., Mao L., Yu G.-T., Bu L.-L., Ma S.-R., Liu B., Gutkind J.S., Kulkarni A.B., Zhang W.-F., Sun Z.-J. (2016). LAG-3 confers poor prognosis and its blockade reshapes antitumor response in head and neck squamous cell carcinoma. OncoImmunology.

[51] Datar I., Sanmamed M.F., Wang J., Henick B.S., Choi J., Badri T., Dong W., Mani N., Toki M., Mejías L.D. (2019). Expression Analysis and Significance of PD-1, LAG-3, and TIM-3 in Human Non–Small Cell Lung Cancer Using Spatially Resolved and Multiparametric Single-Cell Analysis. Clin. Cancer Res..

[52] Wang Y., Dong T., Xuan Q., Zhao H., Qin L., Zhang Q. (2018). Lymphocyte-Activation Gene-3 Expression and Prognostic Value in Neoadjuvant-Treated Triple-Negative Breast Cancer. J. Breast Cancer.

[53] Zhu Z., Ye J., Ma Y., Hua P., Huang Y., Fu X., Li D., Yuan M., Xia Z. (2018). Function of T regulatory type 1 cells is down-regulated and is associated with the clinical presentation of coronary artery disease. Hum. Immunol..

[54] Haudebourg T., Dugast A.-S., Coulon F., Usal C., Triebel F., Vanhove B. (2007). Depletion of LAG-3 Positive Cells in Cardiac Allograft Reveals Their Role in Rejection and Tolerance. Transplantation.

[55] Rodriguez A. (2021). High HDL-Cholesterol Paradox: SCARB1-LAG3-HDL Axis. Curr. Atheroscler. Rep..

[56] Golden D., Kolmakova A., Sura S., Vella A.T., Manichaikul A., Wang X.-Q., Bielinski S.J., Taylor K.D., Chen Y.-D.I., Rich S.S. (2016). Lymphocyte activation gene 3 and coronary artery disease. JCI Insight.

[57] Slevin S.M., Garner L.C., Lahiff C., Tan M., Wang L.M., Ferry H., Greenaway B., Lynch K., Geremia A., Hughes S. (2020). Lymphocyte Activation Gene (LAG)-3 Is Associated With Mucosal Inflammation and Disease Activity in Ulcerative Colitis. J. Crohn’s Colitis.

[58] Do J.-S., Visperas A., Sanogo Y.O., Bechtel J.J., Dvorina N., Kim S., Jang E., Stohlman S.A., Shen B., Fairchild R.L. (2015). An IL-27/Lag3 axis enhances Foxp3+ regulatory T cell–suppressive function and therapeutic efficacy. Mucosal Immunol..

[59] Zhang Z., Duvefelt K., Svensson F., Masterman T., Jonasdottir G., Salter H., Emahazion T., Hellgren D., Falk G., Olsson T. (2005). Two genes encoding immune-regulatory molecules (LAG3 and IL7R) confer susceptibility to multiple sclerosis. Genes Immun..

[60] Delmastro M.M., Styche A.J., Trucco M.M., Workman C.J., Vignali D.A., Piganelli J.D. (2012). Modulation of Redox Balance Leaves Murine Diabetogenic TH1 T Cells “LAG-3-ing” Behind. Diabetes.

[61] Bettini M., Szymczak-Workman A.L., Forbes K., Castellaw A.H., Selby M., Pan X., Drake C.G., Korman A.J., Vignali D.A.A. (2011). Cutting Edge: Accelerated Autoimmune Diabetes in the Absence of LAG-3. J. Immunol..

[62] Doe H.T., Kimura D., Miyakoda M., Kimura K., Akbari M., Yui K. (2016). Expression of PD-1/LAG-3 and cytokine production by CD4+T cells during infection withPlasmodiumparasites. Microbiol Immunol..

[63] Phillips B.L., Mehra S., Ahsan M.H., Selman M., Khader S., Kaushal D. (2014). LAG3 Expression in Active Mycobacterium tuberculosis Infections. Am. J. Pathol..

[64] Graydon C.G., Balasko A.L., Fowke K.R. (2019). Roles, function and relevance of LAG3 in HIV infection. PLOS Pathog..

[65] Jochems S.P., Jacquelin B., Tchitchek N., Busato F., Pichon F., Huot N., Liu Y., Ploquin M.J., Roché E., Cheynier R. (2020). DNA methylation changes in metabolic and immune-regulatory pathways in blood and lymph node CD4 + T cells in response to SIV infections. Clin. Epigenetics.

[66] Wuerdemann N., Pütz K., Eckel H., Jain R., Wittekindt C., Huebbers C.U., Sharma S.J., Langer C., Gattenlöhner S., Büttner R. (2020). LAG-3, TIM-3 and VISTA Expression on Tumor-Infiltrating Lymphocytes in Oropharyngeal Squamous Cell Carcinoma—Potential Biomarkers for Targeted Therapy Concepts. Int. J. Mol. Sci..

[67] Li F.-J., Zhang Y., Jin G.-X., Yao L., Wu D.-Q. (2012). Expression of LAG-3 is coincident with the impaired effector function of HBV-specific CD8+ T cell in HCC patients. Immunol. Lett..

[68] Roy S., Coulon P.-G., Srivastava R., Vahed H., Kim G.J., Walia S.S., Yamada T., Fouladi M.A., Ly V.T., Benmohamed L. (2018). Blockade of LAG-3 Immune Checkpoint Combined With Therapeutic Vaccination Restore the Function of Tissue-Resident Anti-viral CD8+ T Cells and Protect Against Recurrent Ocular Herpes Simplex Infection and Disease. Front. Immunol..

[69] Roy S., Coulon P.-G., Prakash S., Srivastava R., Geertsema R., Dhanushkodi N., Lam C., Nguyen V., Gorospe E., Nguyen A.M. (2019). Blockade of PD-1 and LAG-3 Immune Checkpoints Combined with Vaccination Restores the Function of Antiviral Tissue-Resident CD8 + T RM Cells and Reduces Ocular Herpes Simplex Infection and Disease in HLA Transgenic Rabbits. J. Virol..

[70] Richter K., Agnellini P., Oxenius A. (2009). On the role of the inhibitory receptor LAG-3 in acute and chronic LCMV infection. Int. Immunol..

[71] Anderson A.C., Joller N., Kuchroo V.K. (2016). Lag-3, Tim-3, and TIGIT: Co-inhibitory Receptors with Specialized Functions in Immune Regulation. Immunity.

[72] McLane L.M., Abdel-Hakeem M.S., Wherry E.J. (2019). CD8 T Cell Exhaustion during Chronic Viral Infection and Cancer. Annu. Rev. Immunol..

[73] Chocarro L., Blanco E., Arasanz H., Fernandez-Rubio L., Echaide M., Garnica M., Ramos P., Piñeiro S., Kochan G., Escors D. LAG-3 Role in Infection. Proceedings of the 1st International Electronic Conference on Molecular Sciences: Druggable Targets of Emerging Infectious Disease.

[74] Chocarro L., Blanco E., Arasanz H., Fernandez-Rubio L., Echaide M., Garnica M., Ramos P., Piñeiro S., Kochan G., Escors D. LAG-3 Role in Cardiovascular Diseases. Proceedings of the MOL2NET’22, Conference on Molecular, Biomedical & Computational Sciences and Engineering.

[75] Chocarro L., Blanco E., Arasanz H., Fernandez-Rubio L., Echaide M., Garnica M., Ramos P., Piñeiro S., Kochan G., Escors D. LAG-3 Role in Inflammatory Diseases. Proceedings of the MOL2NET’22, Conference on Molecular, Biomedical & Computational Sciences and Engineering.

[76] Chocarro L., Blanco E., Arasanz H., Fernandez-Rubio L., Echaide M., Garnica M., Ramos P., Piñeiro S., Kochan G., Escors D. LAG-3 Role in Neurological Diseases. Proceedings of the MOL2NET’22, Conference on Molecular, Biomedical & Computational Sciences and Engineering.

[77] Chocarro L., Blanco E., Arasanz H., Fernandez-Rubio L., Bocanegra A., Echaide M., Garnica M., Ramos P., Piñeiro S., Kochan G. Role of the next-generation immune checkpoint LAG-3 in response and resistance to cancer immunotherapy. Proceedings of the MOL2NET’22, Conference on Molecular, Biomedical & Computational Sciences and Engineering.

[78] RELATLIMAB. https://drugs.ncats.io/substance/AF75XOF6W3.

[79] Ascierto P.A., Melero I., Bhatia S., Bono P., Sanborn R.E., Lipson E.J., Callahan M.K., Gajewski T., Gomez-Roca C.A., Hodi F.S. (2017). Initial efficacy of anti-lymphocyte activation gene-3 (anti–LAG-3; BMS-986016) in combination with nivolumab (nivo) in pts with melanoma (MEL) previously treated with anti–PD-1/PD-L1 therapy. J. Clin. Oncol..

[80] Lipson E., Long G., Tawbi H., Schadendorf D., Atkinson V., Maurer M., Simonsen K., Harbison C., Hodi F. (2018). CA224-047: A randomized, double-blind, phase II/III study of relatlimab (anti–LAG-3) in combination with nivolumab (anti–PD-1) versus nivolumab alone in previously untreated metastatic or unresectable melanoma. Ann. Oncol..

[81] Ascierto P.A., Bono P., Bhatia S., Melero I., Nyakas M.S., Svane I.-M., Larkin J., Gomez-Roca C., Schadendorf D., Dummer R. (2017). Efficacy of BMS-986016, a monoclonal antibody that targets lymphocyte activation gene-3 (LAG-3), in combination with nivolumab in pts with melanoma who progressed during prior anti–PD-1/PD-L1 therapy (mel prior IO) in all-comer and biomarker-enriched populations. Ann. Oncol..

[82] Sordo-Bahamonde C., Lorenzo-Herrero S., González-Rodríguez A.P., Payer Á.R., González-García E., López-Soto A., Gonzalez S. (2021). LAG-3 Blockade with Relatlimab (BMS-986016) Restores Anti-Leukemic Responses in Chronic Lymphocytic Leukemia. Cancers.

[83] Clinical Trials Register—Search for 2018-003278-28. https://www.clinicaltrialsregister.eu/ctr-search/search?query=2018-003278-28.

[84] Ellis J., Marks D.J., Srinivasan N., Barrett C., Hopkins T.G., Richards A., Fuhr R., Albayaty M., Coenen M., Liefaard L. (2020). Depletion of LAG-3 + T Cells Translated to Pharmacology and Improvement in Psoriasis Disease Activity: A Phase I Randomized Study of mAb GSK2831781. Clin. Pharmacol. Ther..

[85] Savitsky D., Ward R., Riordan C., Mundt C., Jennings S., Connolly J., Findeis M., Sanicola M., Underwood D., Nastri H. (2018). Abstract 3819: INCAGN02385 is an antagonist antibody targeting the co-inhibitory receptor LAG-3 for the treatment of human malignancies. Cancer Res..

[86] IERAMILIMAB. https://drugs.ncats.io/substance/OI8P0SFD4R.

[87] Lin C.-C., Garralda E., Schöffski P., Hong D., Siu L., Martin M., Maur M., Hui R., Soo R., Chiu J. (2020). 387 A Phase II, multicenter study of the safety and efficacy of LAG525 in combination with spartalizumab in patients with advanced malignancies. J. Immunother. Cancer.

[88] Uboha N.V., Milhem M.M., Kovacs C., Amin A., Magley A., Das Purkayastha D., Piha-Paul S.A. (2019). Phase II study of spartalizumab (PDR001) and LAG525 in advanced solid tumors and hematologic malignancies. J. Clin. Oncol..

[89] Hong D.S., Schoffski P., Calvo A., Sarantopoulos J., De Olza M.O., Carvajal R.D., Prawira A., Kyi C., Esaki T., Akerley W.L. (2018). Phase I/II study of LAG525 ± spartalizumab (PDR001) in patients (pts) with advanced malignancies. J. Clin. Oncol..

[90] Garralda E., Sukari A., Lakhani N.J., Patnaik A., Lou Y., Im S.-A., Golan T., Geva R., Wermke M., De Miguel M. (2021). A phase 1 first-in-human study of the anti-LAG-3 antibody MK4280 (favezelimab) plus pembrolizumab in previously treated, advanced microsatellite stable colorectal cancer. J. Clin. Oncol..

[91] Gregory G.P., Zinzani P.L., Palcza J., Healy J.A., Orlowski R.J., Nahar A., Armand P. (2019). Abstract CT106: Anti-LAG-3 antibody MK-4280 in combination with pembrolizumab for the treatment of hematologic malignancies: A Phase I/II study. Cancer Res..

[92] FAVEZELIMAB. https://drugs.ncats.io/substance/H1396W7D1H.

[93] Burova E., Hermann A., Dai J., Ullman E., Halasz G., Potocky T., Hong S., Liu M., Allbritton O., Woodruff A. (2019). Preclinical Development of the Anti-LAG-3 Antibody REGN3767: Characterization and Activity in Combination with the Anti-PD-1 Antibody Cemiplimab in Human PD-1xLAG-3–Knockin Mice. Mol. Cancer Ther..

[94] FIANLIMAB. https://drugs.ncats.io/substance/OX5LRQ5H6K.

[95] Hamid O., Wang D., Kim T.M., Kim S.-W., Lakhani N.J., Johnson M.L., Groisberg R., Papadopoulos K.P., Kaczmar J.M., Middleton M.R. (2021). Clinical activity of fianlimab (REGN3767), a human anti-LAG-3 monoclonal antibody, combined with cemiplimab (anti-PD-1) in patients (pts) with advanced melanoma. J. Clin. Oncol..

[96] Nanda R., Liu M.C., Yau C., Shatsky R., Pusztai L., Wallace A., Chien A.J., Forero-Torres A., Ellis E., Han H. (2020). Effect of Pembrolizumab Plus Neoadjuvant Chemotherapy on Pathologic Complete Response in Women With Early-Stage Breast Cancer: An Analysis of the Ongoing Phase 2 Adaptively Randomized I-SPY2 Trial. JAMA Oncol..

[97] Papadopoulos K.P., Lakhani N.J., Johnson M.L., Park H., Wang D., Yap T., Dowlati A., Maki R.G., Lynce F., Ulahannan S.V. (2019). First-in-human study of REGN3767 (R3767), a human LAG-3 monoclonal antibody (mAb), ± cemiplimab in patients (pts) with advanced malignancies. J. Clin. Oncol..

[98] Spreafico A., Janku F., Rodon J.A., Tolcher A.W., Chandana S.R., Oliva M., Musalli S., Knauss L., Kragh L., Alifrangis L. (2019). A phase I study of Sym021, an anti-PD-1 antibody (Ab), alone and in combination with Sym022 (anti-LAG-3) or Sym023 (anti-TIM-3). Ann. Oncol..

[99] Lakhani N., Spreafico A., Tolcher A., Rodon J., Janku F., Chandana S., Oliva M., Sharma M., Abdul-Karim R., Hansen U. (2020). 1019O Phase I studies of Sym021, an anti-PD-1 antibody, alone and in combination with Sym022 (anti-LAG-3) or Sym023 (anti-TIM-3). Ann. Oncol..

[100] Ghosh S., Sharma G., Travers J., Kumar S., Choi J., Jun H.T., Kehry M., Ramaswamy S., Jenkins D. (2019). TSR-033, a novel therapeutic antibody targeting LAG-3, enhances T-cell function and the activity of PD-1 blockade in vitro and in vivo. Mol. Cancer Ther..

[101] Park E., Kim H., Sung E., Jung U., Hong Y., Lee H., Ko M., Park Y., Park C.K., Kim S.J. (2021). Abstract 1633: ABL501, PD-L1 x LAG-3, a bispecific antibody promotes enhanced human T cell activation through targeting simultaneously two immune checkpoint inhibitors, LAG-3 and PD-L1. Cancer Res..

[102] Sung E., Ko M., Won J.-Y., Jo Y., Park E., Kim H., Choi E., Jung U.-J., Jeon J., Kim Y. (2022). LAG-3xPD-L1 bispecific antibody potentiates antitumor responses of T cells through dendritic cell activation. Mol. Ther..

[103] Jiang H., Ni H., Zhang P., Guo X., Wu M., Shen H., Wang J., Wu W., Wu Z., Ding J. (2021). PD-L1/LAG-3 bispecific antibody enhances tumor-specific immunity. OncoImmunology.

[104] Powderly J.D., Hurwitz H., Ryan D.P., Laheru D.A., Pandya N.B., Lohr J., Moore P.A., Bonvini E., Wigginton J.M., Crocenzi T.S. (2016). A phase 1, first-in-human, open label, dose escalation study of MGD007, a humanized gpA33 × CD3 DART molecule, in patients with relapsed/refractory metastatic colorectal carcinoma. J. Clin. Oncol..

[105] Hedvat M., Bonzon C., Bernett M.J., Moore G.L., Avery K., Rashid R., Nisthal A., Schubert S., Varma R., Lee S.-H. (2018). Abstract 2784: Simultaneous checkpoint-checkpoint or checkpoint-costimulatory receptor targeting with bispecific antibodies promotes enhanced human T cell activation. Cancer Res..

[106] Everett K.L., Kraman M., Wollerton F.P., Zimarino C., Kmiecik K., Gaspar M., Pechouckova S., Allen N.L., Doody J.F., Tuna M. (2018). Generation of Fcabs targeting human and murine LAG-3 as building blocks for novel bispecific antibody therapeutics. Methods.

[107] Kraman M., Faroudi M., Allen N.L., Kmiecik K., Gliddon D., Seal C., Koers A., Wydro M.M., Batey S., Winnewisser J. (2020). FS118, a Bispecific Antibody Targeting LAG-3 and PD-L1, Enhances T-Cell Activation Resulting in Potent Antitumor Activity. Clin. Cancer Res..

[108] Yap T., Wong D., Hu-Lieskovan S., Papadopoulos K., Morrow M., Grabowska U., Gliddon D., Holz J.-B., LoRusso P. (2020). 395 A first-in-human study of FS118, a tetravalent bispecific antibody targeting LAG-3 and PD-L1, in patients with advanced cancer and resistance to PD-(L)1 therapy. J. ImmunoTherapy Cancer.

[109] Yap T., Papadopoulos K.P., Lorusso P., Wong D.J., Hu-Lieskovan S., Holz J.-B. (2019). A first-in-human phase I study of FS118, an anti-LAG-3/PD-L1 bispecific antibody in patients with solid tumors that have progressed on prior PD-1/PD-L1 therapy. J. Clin. Oncol..

[110] Crescendo Biologics and Cancer Research UK Sign Clinical Development Partnership to Develop CB213, a Novel Bispecific Humabody^®^ Therapeutic | Business Wire. https://www.businesswire.com/news/home/20200505005080/en/Crescendo-Biologics-and-Cancer-Research-UK-sign-Clinical-Development-Partnership-to-develop-CB213-a-novel-bispecific-Humabody%C2%AE-therapeutic.

[111] Edwards C.J., Sette A., Cox C., Di Fiore B., Wyre C., Sydoruk D., Yadin D., Hayes P., Stelter S., Bartlett P.D. (2021). The multi-specific VH-based Humabody CB213 co-targets PD1 and LAG3 on T cells to promote anti-tumour activity. Br. J. Cancer.

[112] Haftcheshmeh S.M., Zamani P., Mashreghi M., Nikpoor A.R., Tavakkol-Afshari J., Jaafari M.R. (2020). Immunoliposomes bearing lymphocyte activation gene 3 fusion protein and P5 peptide: A novel vaccine for breast cancer. Biotechnol. Prog..

[113] Cappello P., Triebel F., Iezzi M., Caorsi C., Quaglino E., Lollini P.L., Amici A., Di Carlo E., Musiani P., Giovarelli M. (2003). LAG-3 Enables DNA Vaccination to Persistently Prevent Mammary Carcinogenesis in HER-2/neu Transgenic BALB/c. Cancer Res..

[114] Fougeray S., Brignone C., Triebel F. (2006). A soluble LAG-3 protein as an immunopotentiator for therapeutic vaccines: Preclinical evaluation of IMP321. Vaccine.

[115] Andreae S., Piras F., Burdin N., Triebel F. (2002). Maturation and Activation of Dendritic Cells Induced by Lymphocyte Activation Gene-3 (CD223). J. Immunol..

[116] Brignone C., Grygar C., Marcu M., Schäkel K., Triebel F. (2007). A soluble form of lymphocyte activation gene-3 (IMP321) induces activation of a large range of human effector cytotoxic cells. J. Immunol..

[117] El Mir S., Triebel F. (2000). A Soluble Lymphocyte Activation Gene-3 Molecule Used as a Vaccine Adjuvant Elicits Greater Humoral and Cellular Immune Responses to Both Particulate and Soluble Antigens. J. Immunol..

[118] Brignone C., Grygar C., Marcu M., Perrin G., Triebel F. (2007). IMP321 (sLAG-3), an immunopotentiator for T cell responses against a HBsAg antigen in healthy adults: A single blind randomised controlled phase I study. J. Immune Based Ther. Vaccines.

[119] Brignone C., Grygar C., Marcu M., Perrin G., Triebel F. (2007). IMP321 (sLAG-3) safety and T cell response potentiation using an influenza vaccine as a model antigen: A single-blind phase I study. Vaccine.

[120] Casati C., Camisaschi C., Rini F., Arienti F., Rivoltini L., Triebel F., Parmiani G., Castelli C. (2006). Soluble Human LAG-3 Molecule Amplifies the In vitro Generation of Type 1 Tumor-Specific Immunity. Cancer Res..

[121] Advanced Melanoma | OpdualagTM (Nivolumab and Relatlimab-Rmbw). https://www.opdualag.com/.

[122] FDA Approves Opdualag for Unresectable or Metastatic Melanoma. https://www.fda.gov/drugs/resources-information-approved-drugs/fda-approves-opdualag-unresectable-or-metastatic-melanoma.

[123] Official Title of Study: A Randomized, Double-Blind Phase 2/3 Study of Relatlimab Combined with Nivolumab Versus Nivolumab in Participants with Previously Untreated Metastatic or Unresectable Melanoma Protocol(S) CA224-047. https://adisinsight.springer.com/trials/700294223.

[124] Fda and Cder, “Highlights of Prescribing Information”. www.fda.gov/medwatch.

[125] Lipson E.J., Tawbi H.A., Schadendorf D., Ascierto P.A., Matamala L., Castillo Gutierrez E., Rutkowski P., Gogas H., Lao C.D., Janoski de Menezes J. (2021). Relatlimab (RELA) plus nivolumab (NIVO) versus NIVO in first-line advanced melanoma: Primary phase III results from RELATIVITY-047 (CA224-047). J. Clin. Oncol..

[126] Long G.V., Hodi F.S., Lipson E.J., Schadendorf D., Ascierto P.A., Matamala L., Salman P., Gutiérrez E.C., Rutkowski P., Gogas H. (2022). Relatlimab and nivolumab versus nivolumab in previously untreated metastatic or unresectable melanoma: Overall survival and response rates from RELATIVITY-047 (CA224-047). J. Clin. Oncol..

[127] Tawbi H.A., Schadendorf D., Lipson E.J., Ascierto P.A., Matamala L., Gutiérrez E.C., Rutkowski P., Gogas H.J., Lao C.D., De Menezes J.J. (2022). Relatlimab and Nivolumab versus Nivolumab in Untreated Advanced Melanoma. N. Engl. J. Med..

[128] Wei S.C., Duffy C.R., Allison J.P. (2018). Fundamental Mechanisms of Immune Checkpoint Blockade Therapy. Cancer Discov..

[129] Rotte A., Jin J.Y., Lemaire V. (2018). Mechanistic overview of immune checkpoints to support the rational design of their combinations in cancer immunotherapy. Ann. Oncol..

[130] Marin-Acevedo J.A., Kimbrough E.O., Lou Y. (2021). Next generation of immune checkpoint inhibitors and beyond. J. Hematol. Oncol..

[131] Monney L., Sabatos C.A., Gaglia J.L., Ryu A., Waldner H., Chernova T., Manning S., Greenfield E.A., Coyle A.J., Sobel R.A. (2002). Th1-specific cell surface protein Tim-3 regulates macrophage activation and severity of an autoimmune disease. Nature.

[132] Gao X., Zhu Y., Li G., Huang H., Zhang G., Wang F., Sun J., Yang Q., Zhang X., Lu B. (2012). TIM-3 Expression Characterizes Regulatory T Cells in Tumor Tissues and Is Associated with Lung Cancer Progression. PLoS ONE.

[133] Anderson A.C., Anderson D.E., Bregoli L., Hastings W.D., Kassam N., Lei C., Chandwaskar R., Karman J., Su E.W., Hirashima M. (2007). Promotion of Tissue Inflammation by the Immune Receptor Tim-3 Expressed on Innate Immune Cells. Science.

[134] Ndhlovu L.C., Lopez-Vergès S., Barbour J.D., Jones R.B., Jha A.R., Long B.R., Schoeffler E.C., Fujita T., Nixon D.F., Lanier L.L. (2012). Tim-3 marks human natural killer cell maturation and suppresses cell-mediated cytotoxicity. Blood.

[135] Zhu C., Anderson A.C., Schubart A., Xiong H., Imitola J., Khoury S., Zheng X.X., Strom T.B., Kuchroo V.K. (2005). The Tim-3 ligand galectin-9 negatively regulates T helper type 1 immunity. Nat. Immunol..

[136] Huang Y.-H., Zhu C., Kondo Y., Anderson A.C., Gandhi A., Russell A.F., Dougan S.K., Petersen B.-S., Melum E., Pertel T. (2015). CEACAM1 regulates TIM-3-mediated tolerance and exhaustion. Nature.

[137] Freeman G.J., Casasnovas J.M., Umetsu D.T., DeKruyff R.H. (2010). TIMgenes: A family of cell surface phosphatidylserine receptors that regulate innate and adaptive immunity. Immunol. Rev..

[138] Chiba S., Baghdadi M., Akiba H., Yoshiyama H., Kinoshita I., Dosaka-Akita H., Fujioka Y., Ohba Y., Gorman J.V., Colgan J.D. (2012). Tumor-infiltrating DCs suppress nucleic acid–mediated innate immune responses through interactions between the receptor TIM-3 and the alarmin HMGB1. Nat. Immunol..

[139] Qin S., Dong B., Yi M., Chu Q., Wu K. (2020). Prognostic Values of TIM-3 Expression in Patients With Solid Tumors: A Meta-Analysis and Database Evaluation. Front. Oncol..

[140] Sakuishi K., Apetoh L., Sullivan J.M., Blazar B.R., Kuchroo V.K., Anderson A.C. (2010). Targeting Tim-3 and PD-1 pathways to reverse T cell exhaustion and restore anti-tumor immunity. J. Exp. Med..

[141] Liu J.-F., Wu L., Yang L.-L., Deng W.-W., Mao L., Wu H., Zhang W.-F., Sun Z.-J. (2018). Blockade of TIM3 relieves immunosuppression through reducing regulatory T cells in head and neck cancer. J. Exp. Clin. Cancer Res..

[142] Zhou Q., Munger M., Veenstra R.G., Weigel B.J., Hirashima M., Munn D., Murphy W.J., Azuma M., Anderson A.C., Kuchroo V.K. (2011). Coexpression of Tim-3 and PD-1 identifies a CD8+ T-cell exhaustion phenotype in mice with disseminated acute myelogenous leukemia. Blood.

[143] Liu J., Zhang S., Hu Y., Yang Z., Li J., Liu X., Deng L., Wang Y., Zhang X., Jiang T. (2016). Targeting PD-1 and Tim-3 Pathways to Reverse CD8 T-Cell Exhaustion and Enhance Ex Vivo T-Cell Responses to Autologous Dendritic/Tumor Vaccines. J. Immunother..

[144] Fourcade J., Sun Z., Benallaoua M., Guillaume P., Luescher I.F., Sander C., Kirkwood J.M., Kuchroo V., Zarour H.M. (2010). Upregulation of Tim-3 and PD-1 expression is associated with tumor antigen–specific CD8+ T cell dysfunction in melanoma patients. J. Exp. Med..

[145] Waterhouse P., Penninger J.M., Timms E., Wakeham A., Shahinian A., Lee K.P., Thompson C.B., Griesser H., Mak T.W. (1995). Lymphoproliferative Disorders with Early Lethality in Mice Deficient in Ctla-4. Science.

[146] Nishimura H., Okazaki T., Tanaka Y., Nakatani K., Hara M., Matsumori A., Sasayama S., Mizoguchi A., Hiai H., Minato N. (2001). Autoimmune Dilated Cardiomyopathy in PD-1 Receptor-Deficient Mice. Science.

[147] Nishimura H., Nose M., Hiai H., Minato N., Honjo T. (1999). Development of Lupus-like Autoimmune Diseases by Disruption of the PD-1 Gene Encoding an ITIM Motif-Carrying Immunoreceptor. Immunity.

[148] Murtaza A., Laken H., Correia J.D.S., McNeeley P., Altobell L., Zhang J., Vancutsem P., Wilcoxen K., Jenkins D. (2016). Discovery of TSR-022, a novel, potent anti-human TIM-3 therapeutic antibody. Eur. J. Cancer.

[149] Falchook G.S., Ribas A., Davar D., Eroglu Z., Wang J.S., Luke J.J., Hamilton E.P., Di Pace B., Wang T., Ghosh S. (2022). Phase 1 trial of TIM-3 inhibitor cobolimab monotherapy and in combination with PD-1 inhibitors nivolumab or dostarlimab (AMBER). J. Clin. Oncol..

[150] Lepletier A., Madore J., O’Donnell J.S., Johnston R.L., Li X.-Y., McDonald E., Ahern E., Kuchel A., Eastgate M., Pearson S.-A. (2020). Tumor CD155 Expression Is Associated with Resistance to Anti-PD1 Immunotherapy in Metastatic Melanoma. Clin. Cancer Res..

[151] O’Donnell J.S., Madore J., Li X.-Y., Smyth M.J. (2019). Tumor intrinsic and extrinsic immune functions of CD155. Semin. Cancer Biol..

[152] Johnston R.J., Comps-Agrar L., Hackney J., Yu X., Huseni M., Yang Y., Park S., Javinal V., Chiu H., Irving B. (2014). The Immunoreceptor TIGIT Regulates Antitumor and Antiviral CD8 + T Cell Effector Function. Cancer Cell.

[153] Yu X., Harden K., Gonzalez L.C., Francesco M., Chiang E., A Irving B., Tom I., Ivelja S., Refino C.J., Clark H. (2008). The surface protein TIGIT suppresses T cell activation by promoting the generation of mature immunoregulatory dendritic cells. Nat. Immunol..

[154] Zhang Q., Bi J., Zheng X., Chen Y., Wang H., Wu W., Wang Z., Wu Q., Peng H., Wei H. (2018). Blockade of the checkpoint receptor TIGIT prevents NK cell exhaustion and elicits potent anti-tumor immunity. Nat. Immunol..

[155] Mao L., Xiao Y., Yang Q.-C., Yang S.-C., Yang L.-L., Sun Z.-J. (2021). TIGIT/CD155 blockade enhances anti-PD-L1 therapy in head and neck squamous cell carcinoma by targeting myeloid-derived suppressor cells. Oral Oncol..

[156] Sarhan D., Cichocki F., Zhang B., Yingst A., Spellman S.R., Cooley S., Verneris M.R., Blazar B.R., Miller J.S. (2016). Adaptive NK Cells with Low TIGIT Expression Are Inherently Resistant to Myeloid-Derived Suppressor. Cells Cancer Res..

[157] Kurtulus S., Sakuishi K., Ngiow S.-F., Joller N., Tan D.J., Teng M., Smyth M., Kuchroo V.K., Anderson A.C. (2015). TIGIT predominantly regulates the immune response via regulatory T cells. J. Clin. Investig..

[158] Duan X., Liu J., Cui J., Ma B., Zhou Q., Yang X., Lu Z., Du Y., Su C. (2019). Expression of TIGIT/CD155 and correlations with clinical pathological features in human hepatocellular carcinoma. Mol. Med. Rep..

[159] Xu D., Zhao E., Zhu C., Zhao W., Wang C., Zhang Z., Zhao G. (2020). TIGIT and PD-1 may serve as potential prognostic biomarkers for gastric cancer. Immunobiology.

[160] Stålhammar G., Seregard S., Grossniklaus H.E. (2019). Expression of immune checkpoint receptors Indoleamine 2,3-dioxygenase and T cell Ig and ITIM domain in metastatic versus nonmetastatic choroidal melanoma. Cancer Med..

[161] Lee W.J., Lee Y.J., Choi M.E., Yun K.A., Won C.H., Lee M.W., Choi J.H., Chang S.E. (2019). Expression of lymphocyte-activating gene 3 and T-cell immunoreceptor with immunoglobulin and ITIM domains in cutaneous melanoma and their correlation with programmed cell death 1 expression in tumor-infiltrating lymphocytes. J. Am. Acad. Dermatol..

[162] Guillerey C., Harjunpää H., Carrié N., Kassem S., Teo T., Miles K., Krumeich S., Weulersse M., Cuisinier M., Stannard K. (2018). TIGIT immune checkpoint blockade restores CD8+ T-cell immunity against multiple myeloma. Blood.

[163] Hung A.L., Maxwell R., Theodros D., Belcaid Z., Mathios D., Luksik A.S., Kim E., Wu A., Xia Y., Garzon-Muvdi T. (2018). TIGIT and PD-1 dual checkpoint blockade enhances antitumor immunity and survival in GBM. OncoImmunology.

[164] Banta K.L., Xu X., Chitre A.S., Au-Yeung A., Takahashi C., O’Gorman W.E., Wu T.D., Mittman S., Cubas R., Comps-Agrar L. (2022). Mechanistic convergence of the TIGIT and PD-1 inhibitory pathways necessitates co-blockade to optimize anti-tumor CD8+ T cell responses. Immunity.

[165] Chen X., Xue L., Ding X., Zhang J., Jiang L., Liu S., Hou H., Jiang B., Cheng L., Zhu Q. (2022). An Fc-Competent Anti-Human TIGIT Blocking Antibody Ociperlimab (BGB-A1217) Elicits Strong Immune Responses and Potent Anti-Tumor Efficacy in Pre-Clinical Models. Front. Immunol..

[166] Frentzas S., Meniawy T., Kao S.C.-H., Wang R., Zuo Y., Zheng H., Tan W. (2021). AdvanTIG-105: Phase 1 dose-escalation study of anti-TIGIT monoclonal antibody ociperlimab (BGB-A1217) in combination with tislelizumab in patients with advanced solid tumors. J. Clin. Oncol..

[167] Cho B.C., Abreu D.R., Hussein M., Cobo M., Patel A.J., Secen N., Lee K.H., Massuti B., Hiret S., Yang J.C.H. (2022). Tiragolumab plus atezolizumab versus placebo plus atezolizumab as a first-line treatment for PD-L1-selected non-small-cell lung cancer (CITYSCAPE): Primary and follow-up analyses of a randomised, double-blind, phase 2 study. Lancet Oncol..

[168] (2021). Genentech: Press Releases. https://www.gene.com/media/press-releases/14892/2021-01-04/genentechs-novel-anti-tigit-tiragolumab-.

[169] Rudin C.M., Liu S.V., Lu S., Soo R.A., Hong M.H., Lee J.-S., Bryl M., Dumoulin D.W., Rittmeyer A., Chiu C.-H. (2022). SKYSCRAPER-02: Primary results of a phase III, randomized, double-blind, placebo-controlled study of atezolizumab (atezo) + carboplatin + etoposide (CE) with or without tiragolumab (tira) in patients (pts) with untreated extensive-stage small cell lung cancer (ES-SCLC). J. Clin. Oncol..

[170] Niu J., Maurice-Dror C., Lee D., Kim D.-W., Nagrial A., Voskoboynik M., Chung H., Mileham K., Vaishampayan U., Rasco D. (2021). First-in-human phase 1 study of the anti-TIGIT antibody vibostolimab as monotherapy or with pembrolizumab for advanced solid tumors, including non-small-cell lung cancer. Ann. Oncol..

[171] Hellmann M., Cho B., Juergens R., Cheng Y., De Castro G., Erman M., Bauman J., Takahashi T., Schwarzenberger P., Zhang P. (2021). P14.01 Phase 3 Study of First-Line Pembrolizumab ± Vibostolimab (anti-TIGIT) in Patients With PD-L1—Positive Metastatic NSCLC. J. Thorac. Oncol..

[172] Garni-Wagner B.A., Lee Z.H., Kim Y.-J., Wilde C., Kang C.-Y., Kwon B.S. (1996). 4-1BB Is Expressed on CD45RAhiROhiTransitional T Cell in Humans. Cell. Immunol..

[173] Wortzman M.E., Clouthier D.L., McPherson A.J., Lin G.H.Y., Watts T.H. (2013). The contextual role of TNFR family members in CD8+T-cell control of viral infections. Immunol. Rev..

[174] Tran B., Carvajal R.D., Marabelle A., Patel S.P., Lorusso P.M., Rasmussen E., Juan G., Upreti V.V., Beers C., Ngarmchamnanrith G. (2018). Dose escalation results from a first-in-human, phase 1 study of glucocorticoid-induced TNF receptor–related protein agonist AMG 228 in patients with advanced solid tumors. J. Immunother. Cancer.

[175] Balmanoukian A.S., Infante J.R., Aljumaily R., Naing A., Chintakuntlawar A.V., Rizvi N.A., Ross H.J., Gordon M., Mallinder P.R., Elgeioushi N. (2020). Safety and Clinical Activity of MEDI1873, a Novel GITR Agonist, in Advanced Solid Tumors. Clin. Cancer Res..

[176] Heinhuis K.M., Carlino M., Joerger M., Di Nicola M., Meniawy T., Rottey S., Moreno V., Gazzah A., Delord J.-P., Paz-Ares L. (2020). Safety, Tolerability, and Potential Clinical Activity of a Glucocorticoid-Induced TNF Receptor–Related Protein Agonist Alone or in Combination With Nivolumab for Patients With Advanced Solid Tumors: A Phase 1/2a Dose-Escalation and Cohort-Expansion Clinical Trial. JAMA Oncol..

[177] Geva R., Voskoboynik M., Dobrenkov K., Mayawala K., Gwo J., Wnek R., Chartash E., Long G. (2020). First-in-human phase 1 study of MK-1248, an anti–glucocorticoid-induced tumor necrosis factor receptor agonist monoclonal antibody, as monotherapy or with pembrolizumab in patients with advanced solid tumors. Cancer.

[178] Papadopoulos K.P., Autio K.A., Golan T., Dobrenkov K., Chartash E., Chen Q., Wnek R., Long G.V. (2021). Phase I Study of MK-4166, an Anti-human Glucocorticoid-Induced TNF Receptor Antibody, Alone or with Pembrolizumab in Advanced Solid Tumors. Clin. Cancer Res..

[179] Gutierrez M., Moreno V., Heinhuis K.M., Olszanski A.J., Spreafico A., Ong M., Chu Q.S., Carvajal R.D., Trigo J., Ochoa de Olza M. (2021). OX40 Agonist BMS-986178 Alone or in Combination With Nivolumab and/or Ipilimumab in Patients With Advanced Solid Tumors. Clin. Cancer Res..

[180] Moiseyenko A., Muggia F., Condamine T., Pulini J., Janik J.E., Cho D.C. (2020). Sequential therapy with INCAGN01949 followed by ipilimumab and nivolumab in two patients with advanced ovarian carcinoma. Gynecol. Oncol. Rep..

[181] Kim T.W., Burris H.A., Luken M.J.D.M., Pishvaian M.J., Bang Y.-J., Gordon M., Awada A., Camidge D.R., Hodi F.S., McArthur G.A. (2022). First-In-Human Phase I Study of the OX40 Agonist MOXR0916 in Patients with Advanced Solid Tumors. Clin. Cancer Res..

[182] Postel-Vinay S., Lam V.K., Ros W., Bauer T.M., Hansen A.R., Cho D.C., Hodi F.S., Schellens J.H., Litton J.K., Aspeslagh S. (2020). Abstract CT150: A first-in-human phase I study of the OX40 agonist GSK3174998 (GSK998) +/- pembrolizumab in patients (Pts) with selected advanced solid tumors (ENGAGE-1). Cancer Res..

[183] Duhen R., Ballesteros-Merino C., Frye A.K., Tran E., Rajamanickam V., Chang S.-C., Koguchi Y., Bifulco C.B., Bernard B., Leidner R.S. (2021). Neoadjuvant anti-OX40 (MEDI6469) therapy in patients with head and neck squamous cell carcinoma activates and expands antigen-specific tumor-infiltrating T cells. Nat. Commun..

[184] Glisson B.S., Leidner R.S., Ferris R.L., Powderly J., Rizvi N.A., Keam B., Schneider R., Goel S., Ohr J.P., Burton J. (2020). Safety and Clinical Activity of MEDI0562, a Humanized OX40 Agonist Monoclonal Antibody, in Adult Patients with Advanced Solid Tumors. Clin. Cancer Res..

[185] El-Khoueiry A.B., Spano J.-P., Angevin E., Doi T., Bullock A.J., Harris W.P., Hamid O., Gougis P., Forgie A., Yang W. (2020). Analysis of OX40 agonist antibody (PF-04518600) in patients with hepatocellular carcinoma. J. Clin. Oncol..

[186] Shapira-Frommer R., van Dongen M.G., Dobrenkov K., Chartash E., Liu F., Li C., Wnek R., Patel M. (2020). O83 Phase 1 study of an anti-CD27 agonist as monotherapy and in combination with pembrolizumab in patients with advanced solid tumors. J. ImmunoTherapy Cancer.

[187] Ansell S.M., Flinn I., Taylor M.H., Sikic B.I., Brody J., Nemunaitis J., Feldman A., Hawthorne T.R., Rawls T., Keler T. (2020). Safety and activity of varlilumab, a novel and first-in-class agonist anti-CD27 antibody, for hematologic malignancies. Blood Adv..

[188] Sznol M., Hodi F.S., Margolin K., McDermott D.F., Ernstoff M.S., Kirkwood J.M., Wojtaszek C., Feltquate D., Logan T. (2008). Phase I study of BMS-663513, a fully human anti-CD137 agonist monoclonal antibody, in patients (pts) with advanced cancer (CA). J. Clin. Oncol..

[189] Timmerman J., Herbaux C., Ribrag V., Zelenetz A.D., Houot R., Neelapu S.S., Logan T., Lossos I.S., Urba W., Salles G. (2020). Urelumab alone or in combination with rituximab in patients with relapsed or refractory B-cell lymphoma. Am. J. Hematol..

[190] Segal N.H., Logan T.F., Hodi F.S., McDermott D., Melero I., Hamid O., Schmidt H., Robert C., Chiarion-Sileni V., Ascierto P.A. (2017). Results from an Integrated Safety Analysis of Urelumab, an Agonist Anti-CD137 Monoclonal Antibody. Clin. Cancer Res..

[191] Segal N.H., He A.R., Doi T., Levy R., Bhatia S., Pishvaian M.J., Cesari R., Chen Y., Davis C.B., Huang B. (2018). Phase I Study of Single-Agent Utomilumab (PF-05082566), a 4-1BB/CD137 Agonist, in Patients with Advanced Cancer. Clin. Cancer Res..

[192] Tolcher A.W., Sznol M., Hu-Lieskovan S., Papadopoulos K.P., Patnaik A., Rasco D.W., Di Gravio D., Huang B., Gambhire D., Chen Y. (2017). Phase Ib Study of Utomilumab (PF-05082566), a 4-1BB/CD137 Agonist, in Combination with Pembrolizumab (MK-3475) in Patients with Advanced Solid Tumors. Clin. Cancer Res..

[193] Cohen E.E.W., Pishvaian M.J., Shepard D.R., Wang D., Weiss J., Johnson M.L., Chung C.H., Chen Y., Huang B., Davis C.B. (2019). A phase Ib study of utomilumab (PF-05082566) in combination with mogamulizumab in patients with advanced solid tumors. J. Immunother. Cancer.

[194] Chiappori A., Thompson J., Eskens F., Spano J.-P., Doi T., Hamid O., Diab A., Rizvi N., Hu-Lieskovan S., Ros W. (2020). P860 Results from a combination of OX40 (PF-04518600) and 4–1BB (utomilumab) agonistic antibodies in melanoma and non-small cell lung cancer in a phase 1 dose expansion cohort. J. Immunother. Cancer.

[195] Martin-Orozco N., Li Y., Wang Y., Liu S., Hwu P., Liu Y.-J., Dong C., Radvanyi L. (2010). Melanoma Cells Express ICOS Ligand to Promote the Activation and Expansion of T-Regulatory Cells. Cancer Res..

[196] Khayyamian S., Hutloff A., Büchner K., Gräfe M., Henn V., Kroczek R.A., Mages H.W. (2002). ICOS-ligand, expressed on human endothelial cells, costimulates Th1 and Th2 cytokine secretion by memory CD4 + T cells. Proc. Natl. Acad. Sci. USA.

[197] Han Y., Dong Y., Yang Q., Xu W., Jiang S., Yu Z., Yu K., Zhang S. (2018). Acute Myeloid Leukemia Cells Express ICOS Ligand to Promote the Expansion of Regulatory T Cells. Front. Immunol..

[198] Lee H.-J., Kim S.-N., Jeon M.-S., Yi T., Song S.U. (2017). ICOSL expression in human bone marrow-derived mesenchymal stem cells promotes induction of regulatory T cells. Sci. Rep..

[199] Marinelli O., Nabissi M., Morelli M.B., Torquati L., Amantini C., Santoni G. (2018). ICOS-L as a Potential Therapeutic Target for Cancer Immunotherapy. Curr. Protein Pept. Sci..

[200] Gigoux M., Shang J., Pak Y., Xu M., Choe J., Mak T.W., Suh W.-K. (2009). Inducible costimulator promotes helper T-cell differentiation through phosphoinositide 3-kinase. Proc. Natl. Acad. Sci. USA.

[201] Conrad C., Gregorio J., Wang Y.-H., Ito T., Meller S., Hanabuchi S., Anderson S., Atkinson N., Ramirez P.T., Liu Y.-J. (2012). Plasmacytoid Dendritic Cells Promote Immunosuppression in Ovarian Cancer via ICOS Costimulation of Foxp3+ T-Regulatory Cells. Cancer Res..

[202] Ito T., Yang M., Wang Y.-H., Lande R., Gregorio J., Perng O.A., Qin X.-F., Liu Y.-J., Gilliet M. (2007). Plasmacytoid dendritic cells prime IL-10–producing T regulatory cells by inducible costimulator ligand. J. Exp. Med..

[203] Strauss L., Bergmann C., Szczepanski M.J., Lang S., Kirkwood J.M., Whiteside T.L. (2008). Expression of ICOS on Human Melanoma-Infiltrating CD4+CD25highFoxp3+ T Regulatory Cells: Implications and Impact on Tumor-Mediated Immune Suppression. J. Immunol..

[204] Faget J., Bendriss-Vermare N., Gobert M., Durand I., Olive D., Biota C., Bachelot T., Treilleux I., Goddard-Leon S., Lavergne E. (2012). ICOS-Ligand Expression on Plasmacytoid Dendritic Cells Supports Breast Cancer Progression by Promoting the Accumulation of Immunosuppressive CD4+ T Cells. Cancer Res..

[205] Chavez J.C., Foss F.M., William M.B.M., E Brammer J., Smith S.M., Prica A., Zain J.M., Tuscano J.M., Glenn M., Mehta-Shah N. (2020). A Phase I Study of Anti-ICOS Antibody MEDI-570 for Relapsed/Refractory (R/R) Peripheral T-Cell Lymphoma (PTCL) and Angioimmunoblastic T-Cell Lymphoma (AITL) (NCI-9930). Blood.

[206] Patel M.R., Naing A., Iii H.A.B., Lin C.-C., Curigliano G., Thistlethwaite F., Minchom A.R., Ascierto P.A., De Braud F.G., Cecchini M. (2021). A phase 1/2 open-label study of KY1044, an anti-ICOS antibody with dual mechanism of action, as single agent and in combination with atezolizumab, in adult patients with advanced malignancies. J. Clin. Oncol..

[207] Sainson R.C., Thotakura A.K., Kosmac M., Borhis G., Parveen N., Kimber R., Carvalho J., Henderson S.J., Pryke K.L., Okell T. (2020). An Antibody Targeting ICOS Increases Intratumoral Cytotoxic to Regulatory T-cell Ratio and Induces Tumor Regression. Cancer Immunol. Res..

[208] Hansen A., Bauer T., Moreno V., Maio M., Groenland S., Martin-Liberal J., Gan H., Rischin D., Millward M., Olszanski A. (2018). First in human study with GSK3359609 [GSK609], inducible T cell co-stimulator (ICOS) receptor agonist in patients [Pts] with advanced, solid tumors: Preliminary results from INDUCE-1. Ann. Oncol..

[209] Fan X., Quezada S., Sepulveda M.A., Sharma P., Allison J.P. (2014). Engagement of the ICOS pathway markedly enhances efficacy of CTLA-4 blockade in cancer immunotherapy. J. Exp. Med..

[210] Burris H.A., Callahan M.K., Tolcher A.W., Kummar S., Falchook G.S., Pachynski R.K., Tykodi S.S., Gibney G.T., Seiwert T.Y., Gainor J.F. (2017). Phase 1 safety of ICOS agonist antibody JTX-2011 alone and with nivolumab (nivo) in advanced solid tumors; predicted vs observed pharmacokinetics (PK) in ICONIC. J. Clin. Oncol..

[211] Yap T.A., Burris H.A., Kummar S., Falchook G.S., Pachynski R.K., Lorusso P., Tykodi S.S., Gibney G.T., Gainor J.F., Rahma O.E. (2018). ICONIC: Biologic and clinical activity of first in class ICOS agonist antibody JTX-2011 +/- nivolumab (nivo) in patients (pts) with advanced cancers. J. Clin. Oncol..

[212] Kobziev O., Bulat I., Ostapenko Y., Zvirbule Z., Ursol G., Boyko V., Paramonov V., Hashambhoy-Ramsay Y., Hart C., Harvey C. (2021). Phase 2 study of PD-1 inhibitor JTX-4014 alone and in combination with vopratelimab, an ICOS agonist, in biomarker-selected subjects with metastatic NSCLC after one prior platinum-containing regimen (SELECT). J. Clin. Oncol..

